# circNFIB1 inhibits lymphangiogenesis and lymphatic metastasis via the miR-486-5p/PIK3R1/VEGF-C axis in pancreatic cancer

**DOI:** 10.1186/s12943-020-01205-6

**Published:** 2020-05-04

**Authors:** Yao Kong, Yuting Li, Yuming Luo, Jiang Zhu, Hanhao Zheng, Bowen Gao, Xiaofeng Guo, Zhihua Li, Rufu Chen, Changhao Chen

**Affiliations:** 1 Department of Ultrasound, Sun Yat-sen Memorial Hospital, Guangzhou, Guangdong 510120 People’s Republic of China; 2 Guangdong Provincial Key Laboratory of Malignant Tumor Epigenetics and Gene Regulation, Sun Yat-sen Memorial Hospital, State Key Laboratory of Oncology in South China, Guangzhou, Guangdong 510120 People’s Republic of China; 3 Department of Medical Oncology, Sun Yat-sen Memorial Hospital, 107th Yanjiangxi Road, Yuexiu District, Guangzhou, Guangdong 510120 People’s Republic of China; 4 Department of Pancreatobiliary Surgery, Sun Yat-sen Memorial Hospital, Guangzhou, Guangdong 510120 People’s Republic of China; 5 Department of General Surgery, Guangdong Provincial People’s Hospital, Guangdong Academy of Medical Sciences, Guangzhou, Guangdong 510080 People’s Republic of China; 6 Department of Urology, Sun Yat-sen Memorial Hospital, 107 Yanjiangxi Road, Yuexiu District, Guangzhou, Guangdong 510120 People’s Republic of China

**Keywords:** circNFIB1, PIK3R1, PI3K/Akt signaling pathway, Lymphatic metastasis, Pancreatic cancer

## Abstract

**Background:**

Patients with lymph node (LN)-positive pancreatic ductal adenocarcinoma (PDAC) have extremely poor survival rates. Circular RNAs (circRNAs), a newly discovered type of endogenous noncoding RNAs, have been proposed to mediate the progression of diverse types of tumors. However, the role and underlying regulatory mechanisms of circRNAs in the LN metastasis of PDAC remain unknown.

**Methods:**

Next-generation sequencing was used to identify differentially expressed circRNAs between PDAC and normal adjacent tissues. In vitro and in vivo experiments were conducted to evaluate the functional role of circNFIB1. RNA pulldown and luciferase assays were performed to examine the binding of circNFIB1 and miR-486-5p.

**Results:**

In the present study, we identified that a novel circRNA (circNFIB1, hsa_circ_0086375) was downregulated in PDAC and negatively associated with LN metastasis in PDAC patients. Functionally, circNFIB1 knockdown promoted lymphangiogenesis and LN metastasis of PDAC both in vitro and in vivo. Mechanistically, circNFIB1 functioned as a sponge of miR-486-5p, and partially reversed the effect of miR-486-5p. Moreover, circNFIB1 attenuated the oncogenic effect of miR-486-5p and consequently upregulated PIK3R1 expression, which further downregulated VEGF-C expression through inhibition of the PI3K/Akt pathway, and ultimately suppressed lymphangiogenesis and LN metastasis in PDAC.

**Conclusions:**

Our findings provide novel insight into the underlying mechanism of circRNA-mediated LN metastasis of PDAC and suggest that circNFIB1 may serve as a potential therapeutic target for LN metastasis in PDAC.

**Graphical abstract:**

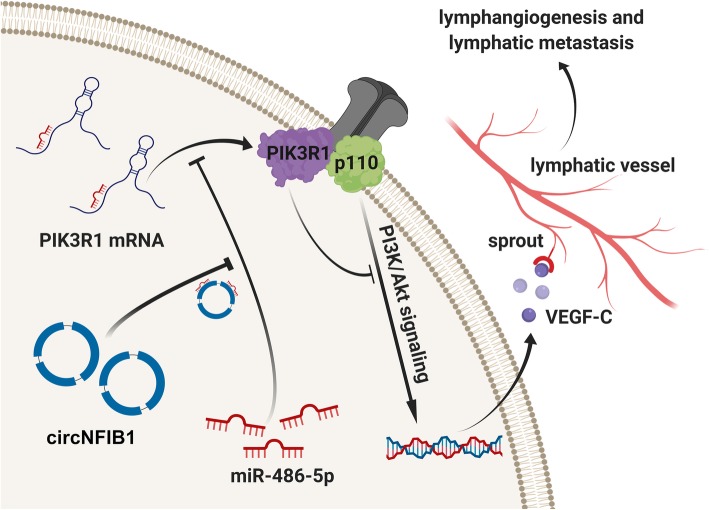

## Introduction

Pancreatic ductal adenocarcinoma (PDAC) is one of the most lethal malignant cancers and currently ranks fourth in cancer-related death worldwide [1–3]. Despite the improved clinical technology, the prognosis of PDAC remains poor and its average five-year survival rate is less than 5% [3, 4]. Previous studies have demonstrated that the poor prognosis of PDAC is predominantly caused by its early invasion of the lymph nodes, which contributes to devasting dissemination into the peripheral tissues and metastasis to distant places [5, 6]. Despite the obvious clinical importance of lymphatic metastasis in PDAC, the mechanisms that lead to PDAC spread via the lymphatic vessels remain largely unknown.

Lymphatic vessels serve as the dissemination routes for cancer cells from the local tumor to the lymph nodes, and subsequently to distant locations via the blood [7]. The expansion of lymphatic vessels, termed lymphangiogenesis, is considered to be the most critical and the rate-limiting step among the processes by which cancer cells metastasize via the LNs [8–10]. Previous studies have demonstrated that lymphangiogenesis is positively regulated by vascular endothelial growth factor-C (VEGF-C) and blocking the secretion of VEGF-C via its inhibitor suppresses the formation of new lymphatic vessels, which subsequently limits the LN metastasis of tumors [11]. Nevertheless, although VEGF-C plays a crucial role in lymphangiogenesis and lymphatic metastasis, little is known of the regulatory mechanism of VEGF-C expression and its induction of lymphangiogenesis in PDAC.

Circular RNAs (circRNAs) represent a type of novel noncoding RNAs with a covalently circular structure arising from the non-canonical splicing of pre-mRNAs. Moreover, circRNAs lack 5′ caps and 3 poly-A tails and are much more stable compared with their linear counterparts since they are resistant to exonuclease [12]. CircRNAs are widely expressed in several cancers and play essential roles in regulating cancer progression [13]. Yang et al. found that circ-ITCH functioned as a tumor suppressor in bladder cancer through the circ-ITCH/miR-17, miR-224/p21, PTEN axis [14]. In addition, a study conducted by Wei et al. demonstrated that circCDYL promoted the malignant transformation of hepatoma by controlling the tumor-initiating properties of hepatocellular carcinoma cells via activating the PI3K-AKT-mTORC1/β-catenin and NOTCH2 pathways [15]. Nevertheless, the specific regulatory mechanisms of circRNAs in the LN metastasis of PDAC have not been fully explored.

MicroRNAs (miRNAs) are a kind of small endogenous non-coding RNAs comprised of 18 ~ 22 nucleotides, which mainly affect the expression of their target genes post-transcriptionally to regulate the progression of human cancers [16]. As a member of miRNA family, miR-486-5p has been reported to function as an oncogene in multiple cancers. Lopez-Bertoni et al. found that the inhibition of miR-486-5p reduced tumor size via a PTEN-dependent mechanism in vivo [17]. Hanna et al. reported that miR-486-5p promoted the proliferation and invasion of alveolar rhabdomyosarcoma [18]. Although miR-486-5p plays a crucial role in human cancers, the precise regulatory mechanism of miR-486-5p in LN metastasis of PDAC is still not fully investigated.

In this study, we identified a novel circRNA (circNFIB1, has_circ_0086375), which was downregulated in PDAC and negatively associated with LN metastasis in PDAC patients. Functionally, circNFIB1 knockdown was found to promote the lymphangiogenesis and LN metastasis of PDAC. Mechanistically, circNFIB1 functioned as an miR-486-5p sponge, and partially reversed the effect of miR-486-5p. Moreover, circNFIB1 attenuated the oncogenic effect of miR-486-5p, consequently upregulating PIK3R1 expression, which further downregulated VEGF-C expression through inhibition of the PI3K/Akt pathway and ultimately resulted in the suppression of lymphangiogenesis and LN metastasis in PDAC.

## Methods

### Clinical specimens

With the approval of the Protection of Human Subjects Committees at Sun Yat-sen Memorial Hospital, Sun Yat-sen University (Guangzhou, China), paired PDAC tissues and non-cancerous tissues (NATs) were collected from 160 PDAC patients who underwent surgery at Sun Yat-Sen Memorial Hospital, Sun Yat-Sen University from February 2014 to February 2019. Each sample was confirmed by two independent professional pathologists. For RNA extraction, the tissues were immediately snap-frozen in liquid nitrogen and stored at − 80 °C until further investigation. For immunohistochemistry (IHC), the tissues were fixed in 10% (v/v) neutral-buffered formalin followed by 70% ethanol dehydration and then embedded in paraffin. Tumor staging was determined following the instruction of the latest edition of the tumor node metastasis (TNM) system of the American Joint Committee. Written informed consent was obtained from all the patients before performing the study. The detailed clinicopathological characteristics of the patients are summarized in Additional file 1.

### Cell lines and cell culture

The human PDAC cell lines (PANC1, Capan-2, SW1990) and human pancreatic ductal endothelial cells (HPDE) were purchased from the American Type Culture Collection (ATCC, Manassas, VA). The PANC-1, Capan-2 and SW1990 cell lines were maintained in high glucose Dulbecco’s Modified Eagle’s Medium (DMEM, Gibco, USA), supplemented with 10% FBS (BI, Israel). The HPDE cell line was cultured in RPMI 1640 (Gibco, USA), supplemented with 10% FBS. Human lymphatic endothelial cells (HLECs) were obtained from the ATCC and maintained in the Endothelial Cell Medium (ECM, Gibco, USA). All cell lines were cultured under a humidified atmosphere containing 5% CO_2_.

### Next-generation sequencing

Total RNA was isolated using TRIzol reagent. DNase I was applied to eliminate the genomic DNA and Ribo-Zero rRNA Removal Kits (MRZMB126, Epicentre) was applied to deplete ribosomal RNA. We then purified Poly(A) + RNA with oligo [4] magnetic beads and fragmented it into short sequences. The quality of the purified RNA was measured with a BioAnalyzer 2100 (AgilenN Technology, Santa Clara, CA). The library was prepared in accordance with the instructions of the TruSeq Stranded mRNA LT 11 Sample Prep Kit (Cat. No.15032612, Illumina). Each library was sequenced on an Illumina HiSeq2500 in 125PE mode 13 (Illumina, San Diego, CA, USA).

### RNA pull-down assay

For the preparation of streptavidin magnetic beads, the beads were washed three times with an RNase-free lysis buffer, and blocked with 10 μg/μL BSA and yeast tRNA on a rotator at 4 °C for 3 h. The blocked beads were incubated with a biotinylated circNFIB1 probe or oligo probe at 25 °C for 2 h. Later, approximately 2 × 10^7^ PANC1 and Capan-2 cells were harvested and fixed with 1% formaldehyde before being lysed in lysis buffer and incubated with prepared pull-down probes, 50 μL of the cell lysate was collected as the input. The pull-down complexes were harvested and tested by quantitative real-time PCR (qRT-PCR). The probes used in RNA pull-down assay were list in Additional file 2.

### Popliteal LN metastasis study

All animal experiments were conducted following the guidelines approved by the Institutional Animal Care and Use Committee of Sun Yat-sen Memorial Hospital, Sun Yat-sen University. four to five weeks of age, 18 g - 20 g female BALB/c nude mice were purchased from the Experimental Animal Center. Six mice were included in each group, and 100 μL PBS suspension of 5 × 10^6^ PANC-1 cells transduced with sh-circNFIB1-luc or sh-NC-luc were incubated into the footpads of the mice. The mice were imaged with an IVIS Spectrum Imaging System (PerkinElmer) to monitor lymphatic metastasis. When the tumors reached 200 mm^3^, the primary tumor and popliteal LNs were enucleated, embedded in paraffin, and analyzed with an anti-LYVE-1 antibody by IHC. Images were captured with a Nikon Eclipse 80i system with NIS-Elements software (Nikon, Japan).

### Statistical analysis

All quantitative data are presented as the mean ± standard deviations from at least three independent experiments. The cumulative survival time was calculated using the Kaplan-Meier method and analyzed with a log-rank test. A multivariate Cox proportional hazards model was used to estimate the adjusted hazard ratios and 95% confidence intervals, as well as identify independent prognostic factors. Chi-square tests (*χ*^2^ tests) were used to assess the relationships between non-parametric variables, and two-tailed Student’s *t*-test or one-way ANOVA was used to evaluate the relationship between parametric variables. The threshold for statistical significance was set at *p* < 0.05. For multiple comparisons, the *p*-value was corrected by using Bonferroni correction. All statistical tests were performed with IBM. (Version 19.0.; IBM Corp., Armonk, NY).

### Further applied methods

Additional lentivirus infection and cell transfection, qRT-PCR, HLECs tube formation assay and Transwell assay, subcellular fractionation assay, fluorescence in situ hybridization (FISH), Colocalization of circNFIB1 and miR-486a-5p, luciferase activity assay, Western Blotting, IHC, ELISA-based quantification of secreted VEGF-C, gel electrophoresis analysis are further described in the Additional file 3.

## Results

### Identification and the characteristics of circNFIB1 in PDAC

**Fig. 1:**
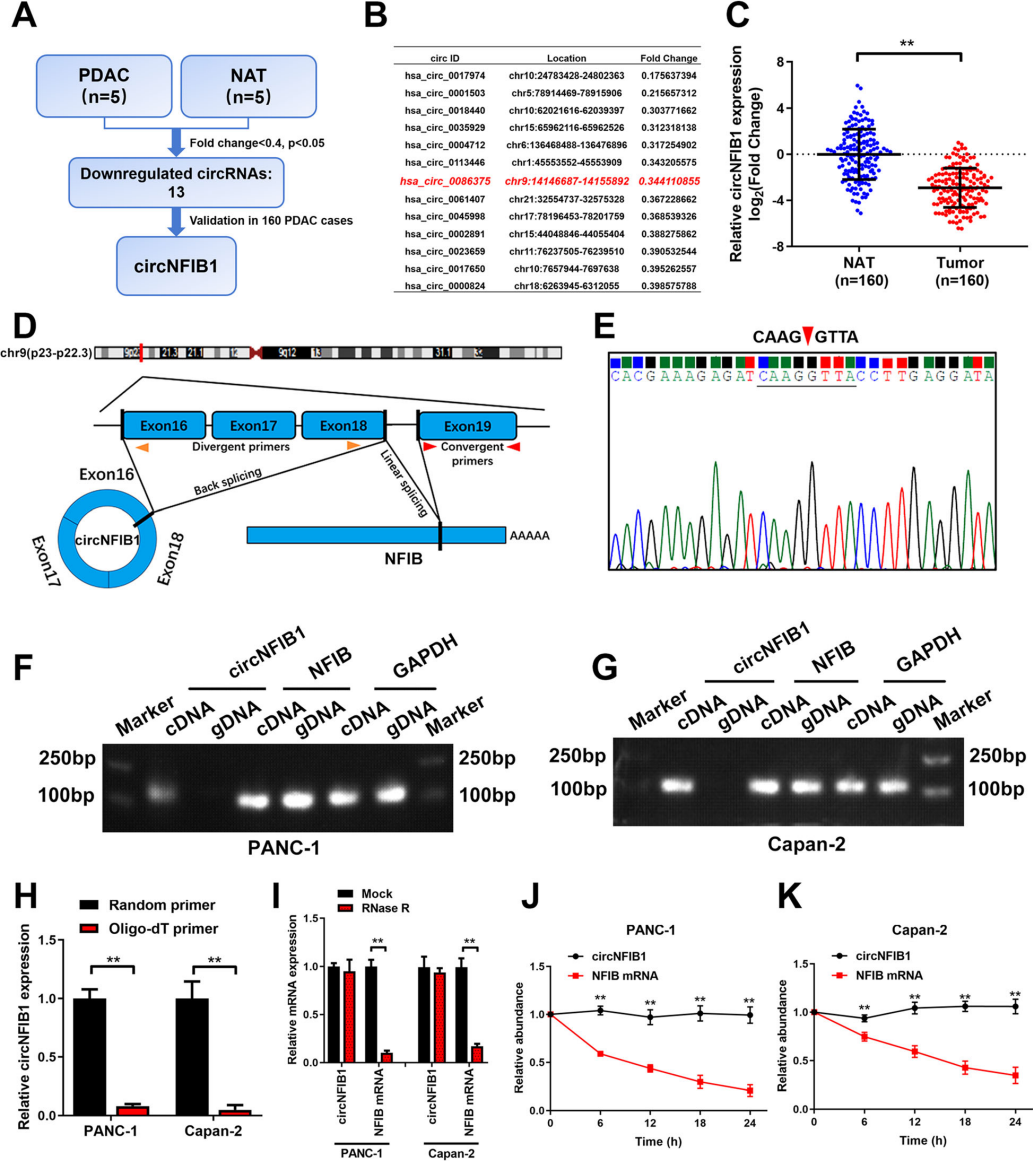
The identification and characterization of circNFIB1 in PDAC. **a** Schematic illustration of the identification of circRNAs downregulated in PDAC tissues compared with NATs. **b** The downregulated circRNAs in PDAC identified from NGS were shown. **c** qRT-PCR analysis of circNFIB1 expression in PDAC tissues (*n* = 160) paired with NATs (*n* = 160). The nonparametric Mann-Whitney U test was used to compare different groups. **d** Schematic illustrations showed the genomic loci of circNFIB1. circNFIB1 was produced by exons 16 to 18 of NFIB. **e** The back-splice junction of circNFIB1 was identified by Sanger sequencing. **f and g** PCR analysis for circNFIB1 and NFIB in the cDNA and gDNA of PANC-1 (**f**) or Capan-2 (**g**) cells. GAPDH was used as NC. **h** qRT-PCR analysis of circNFIB1 expression using random primers or oligo-dT primers. **i** circNFIB1 and NFIB expression in PDAC cells treated with or without RNase R was assessed by qRT-PCR. **j and k** qRT-PCR analysis of circNFIB1 and NFIB mRNA in PANC-1 (j) or Capan-2 (**k**) cells treated with actinomycin D at the indicated time points. Significance level was assessed using two-tailed Student *t*-tests. Figures with error bars showed the standard deviations of three independent experiments. *p < 0.05 and **p < 0.01

To identify the essential circRNAs that contribute to the progression of PDAC, next-generation sequencing was performed to analyze five pairs of PDAC tissues and the corresponding normal adjacent tissues (NATs)(GSE136569). 13 circRNAs were differentially downregulated (fold-change < 0.4 and *P* < 0.05) in PDAC tissues and further validated in a larger cohort of 160-case of PDAC tissues and paired NATs by qRT-PCR analysis (Fig. 1a, b). We then focused on circNFIB1 (hsa_circ_0086375), which was expressed at low level in PDAC tissues, for further study (Fig. 1c).

Since circRNAs differ from their counterpart genes regarding their secondary structure, we next evaluated the circular structure of circNFIB1, which was aroused from exons 16 to 18 of the NFIB gene (chr9: 14146687–14,155,892) (Fig. 1d). Sanger sequencing validated the back-spliced junction of circNFIB1 (Fig. 1e), which was consistent with the sequences obtained from circBase, and the distinct product of circNFIB1 could only be amplified from the cDNA but not from the gDNA (Fig. 1f, g). Furthermore, we found that circNFIB1 expression was significantly decreased when oligo-dT primers were used for reverse transcription compared with the use of random primers, indicating the absence of poly-A tail in circRNA, which in construct, exists in linear RNAs (Fig. 1h). Using treatment with RNase R, an exoribonuclease which could only degrade linear mRNA, NFIB mRNA expression dramatically decreased, whereas circNFIB1 expression remained unaffected, further confirming that circNFIB1 consisted of a closed circular structure (Fig. 1i). Since RNAs that exist in a circular form are more stable than the linear form, we further analyzed the stability of circNFIB1. We observed a longer half-life of circNFIB1 compared with that of NFIB mRNA in PDAC cells treated with Actinomycin D, a transcription inhibitor (Fig. 1j, k). Taken together, these results indicate that circNFIB1 is a highly stable circRNA derives from exons 16 to 18 of the NFIB gene locus.

### circNFIB1 is negatively associated with LN metastasis in PDAC patients

**Fig. 2:**
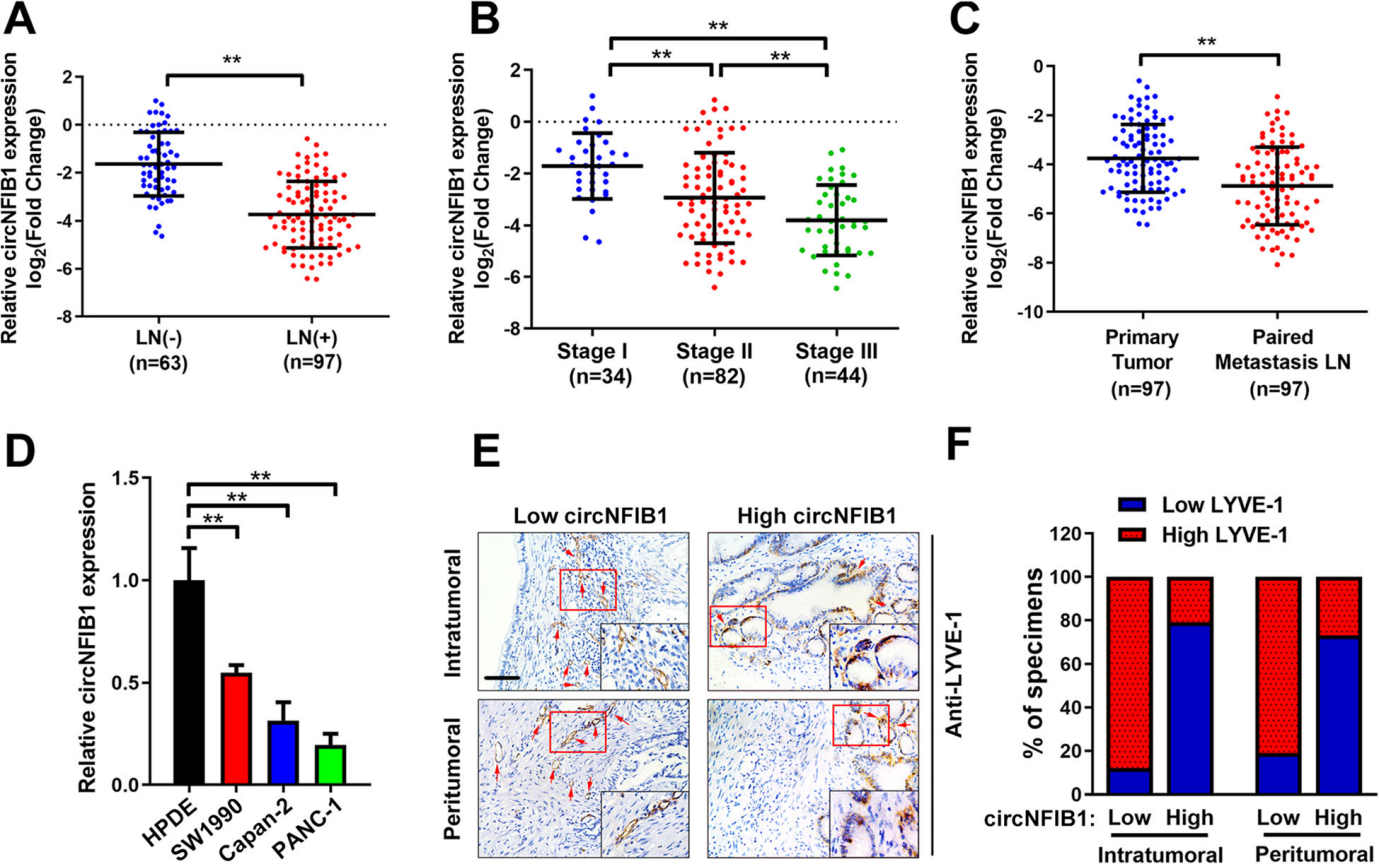
CircNFIB1 is downregulated in PDAC and negatively correlates with LN metastasis. **a and b** circNFIB1 expression in human PDAC tissues (*n* = 160) according to LN status (**a**) and tumor stages (**b**) was assessed by qRT-PCR. The nonparametric Mann-Whitney U test was used to compare different groups. **c** qRT-PCR analyzed the expression of circNFIB1 in primary PDAC tissues and paired metastatic LNs (*n* = 160). The nonparametric Mann-Whitney U test was used to compare different groups. **d** The expression of circNFIB1 in PDAC and normal pancreatic ductal epithelial cell lines was detected by qRT-PCR. **e and f** Representative images (**e**) and percentages (**f**) for IHC staining showed that LYVE-1 indicated the lymphatic vessel density in PDAC tissues with differential circNFIB1 expression. Scale bars: 50 μm. Significance level was assessed using one-way ANOVA followed by Dunnett’s tests for multiple comparisons. Figures with error bars showed the standard deviations of three independent experiments. *p < 0.05 and **p < 0.01

To study the role of circNFIB1 in PDAC, we analyzed circNFIB1 expression in a cohort of 160 PDAC patients. We found that patients with lymphatic metastasis and a highly pathological TMN stage had lower circNFIB1 expression (Fig. 2a, b and Additional file 1: Table S1). In addition, qRT-PCR analysis showed that there were lower levels of circNFIB1 in metastatic tumor cells in the LNs compared with the paired primary tumors (Fig. 2c). Consistent with the results obtained from clinical samples, a lower expression of circNFIB1 was detected in PDAC cells compared with HPDE (Fig. 2d). Moreover, IHC analysis showed a negative correlation between circNFIB1 and lymphatic vessel density (Fig. 2e, f). Together, our findings suggest that circNFIB1 is essential for the inhibition of the LN metastasis of PDAC.

### circNFIB1 inhibits lymphangiogenesis in vitro

**Fig. 3:**
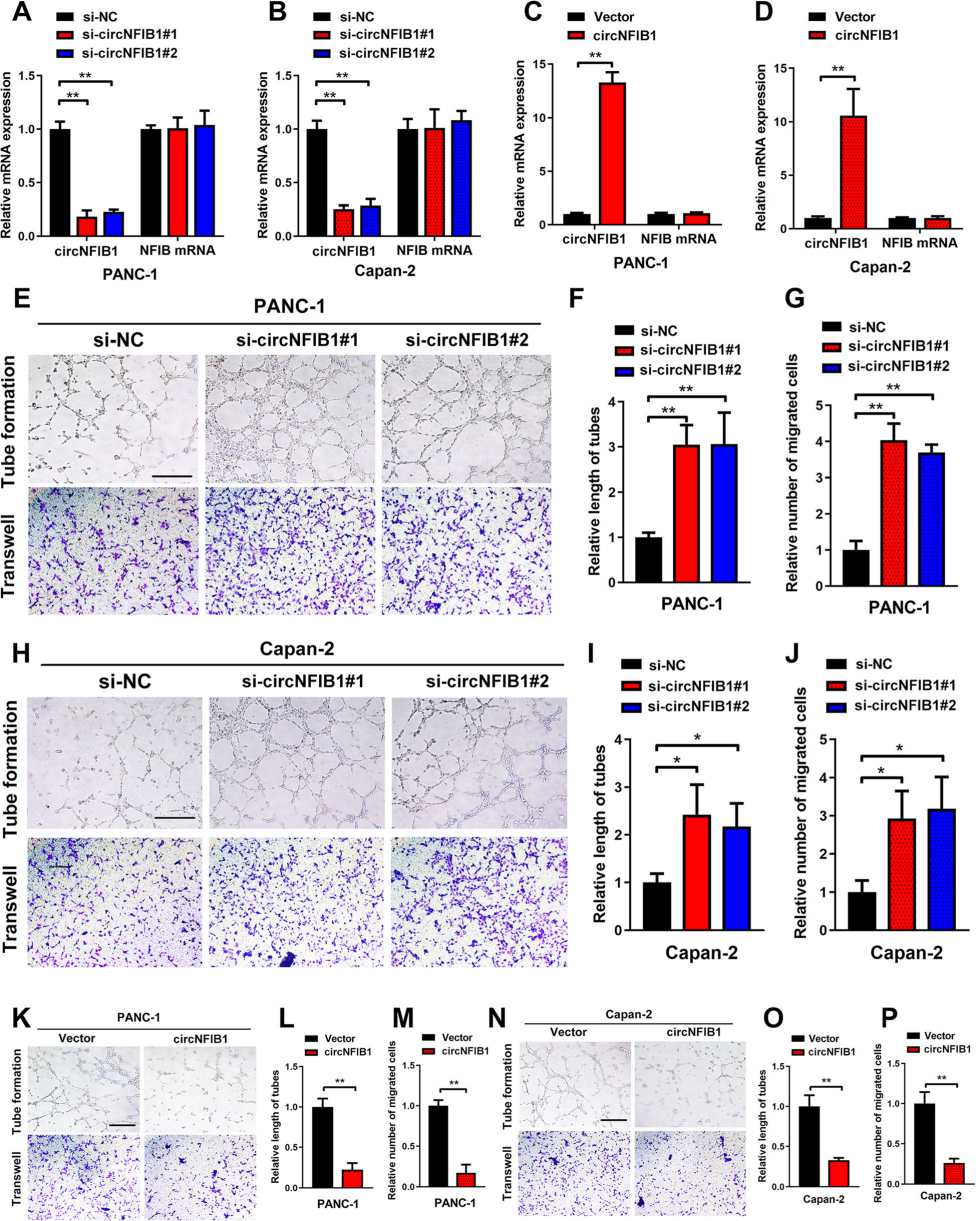
CircNFIB1 suppresses lymphangiogenesis in vitro. **a-d** qRT-PCR analysis of circNFIB1 and NFIB expression following circNFIB1-silencing (**a and b**), circNFIB1 overexpression (**c and d**) or corresponding control PDAC cells. **e-j** Representative images (**e and h**) and histogram analysis of tube formation (**f and i**) and Transwell (**g and j**) assays by HLECs treated with conditioned medium from circNFIB1-silencing or control PDAC cells. Scale bar: 100 μm. **k-p** Representative images (**k and n**) and histogram analysis of tube formation (**l and o**) and Transwell (**m and p**) assays by HLECs treated with conditioned medium from circNFIB1-overexpressing or control PDAC cells. Scale bar: 100 μm. Significance level was assessed using two-tailed Student *t*-tests and one-way ANOVA followed by Dunnett’s tests for multiple comparisons. Figures with error bars showed the standard deviations of three independent experiments. *p < 0.05 and **p < 0.01

Given that lymphangiogenesis is a key factor for LN metastasis, we further explored whether circNFIB1 inhibits lymphangiogenesis in vitro*.* The expression of circNFIB1 was downregulated by transfection with siRNAs that specifically targeted the back-spliced region of circNFIB1 without affecting NFIB expression (Fig. 3a, b). Similarly, circNFIB1 was successfully overexpressed by transfection with circNFIB1 plasmids, and no obvious changes were observed with NFIB (Fig. 3c, d). In vitro assays showed that the conditioned media from circNFIB1-silencing PDAC dramatically promoted the tube formation and migration ability of HLECs (Fig. 3e-j). In contrast, conditioned media from circNFIB1-transduced PDAC significantly inhibited the HLEC tube formation and migration compared with the control group (Fig. 3k-p). Moreover, given that enhanced migration ability of cancer cells contributes to LN metastasis, we further examined the effect of circNFIB1 on the migration of PDAC cells. Transwell assays showed that circNFIB1 knockdown promoted the migration ability of PANC-1, Capan-2 and SW1990 cells, suggesting that circNFIB1 inhibited the migration of PDAC cells (Additional file 4: Fig. S1a, b). Together, these results indicate that circNFIB1 suppresses the lymphangiogenesis of PDAC in vitro.

### circNFIB1 suppresses LN metastasis of PDAC in vivo

**Fig. 4:**
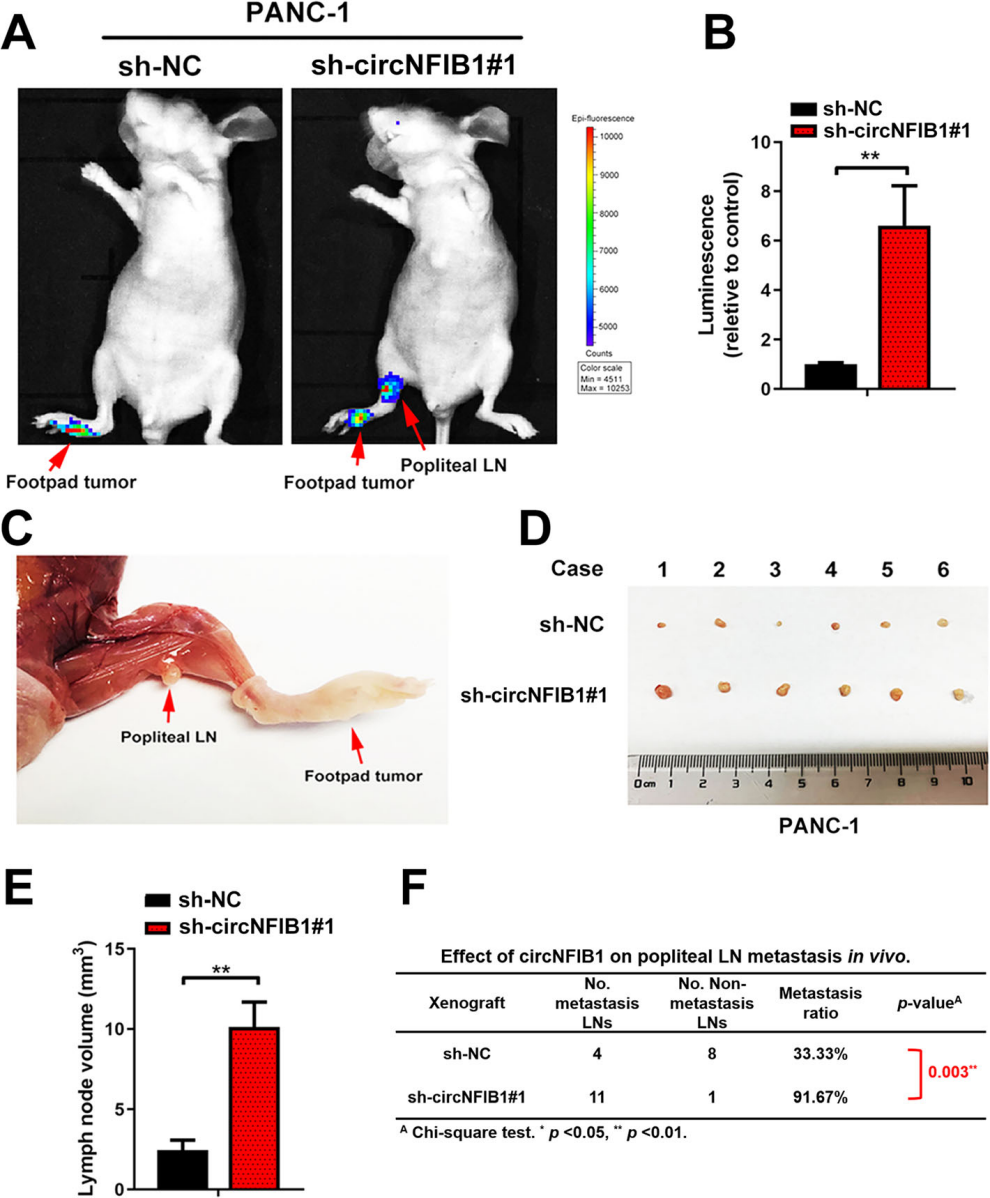
CircNFIB1 inhibits LN metastasis of PDAC in vivo. **a and b** Representative images (**a**) of bioluminescence and histogram analysis (**b**) of popliteal LN metastasis after silencing circNFIB1 (*n* = 12 per group). **c** Representative images for the nude mouse popliteal LN metastasis model. PDAC cells were injected into the footpads of the nude mice and the popliteal LNs were shown. **d** Representative images of enucleated popliteal LNs (*n* = 12 per group). **e** Histogram analysis of the LN volume in the indicated groups. **f** The ratio of popliteal LN metastasis was calculated for all groups (*n* = 12 per group). Significance level was assessed using two-tailed Student *t*-tests and one-way ANOVA followed by Dunnett’s tests for multiple comparisons. Figures with error bars showed the standard deviations of three independent experiments. *p < 0.05 and **p < 0.01

Next, we employed a popliteal LN metastasis model to examine the role of circNFIB1 in the LN metastasis of PDAC in vivo. Briefly, sh-circNFIB1#1/luc- and sh-NC/luc-transfected PDAC cells were inoculated into the footpads of nude mice. The effects of circNFIB1 on LN metastasis were analyzed when the footpad tumor size reached 200 mm^3^. Strikingly, the in vivo imaging showed that circNFIB1 knockdown significantly promoted the popliteal LN metastasis of PDAC cells (Fig. 4a, b). The volume of the popliteal LN was larger in the sh-circNFIB1#1 group compared with the control (Fig. 4c-e). Moreover, sh-circNFIB1#1 group exhibited higher LN metastatic rate than the control (Fig. 4f). Additionally, we found that the primary tumor size in sh-NC group was lower than the sh-circNFIB1#1 group at the same time point, suggesting that circNFIB1 suppressed the tumorigenesis of PDAC (Additional file 4: Fig. S1c). Collectively, our results indicate that circNFIB1 inhibits the LN metastasis of PDAC in vivo.

### circNFIB1 functions as a miR-486-5p sponge in PDAC cells

**Fig. 5:**
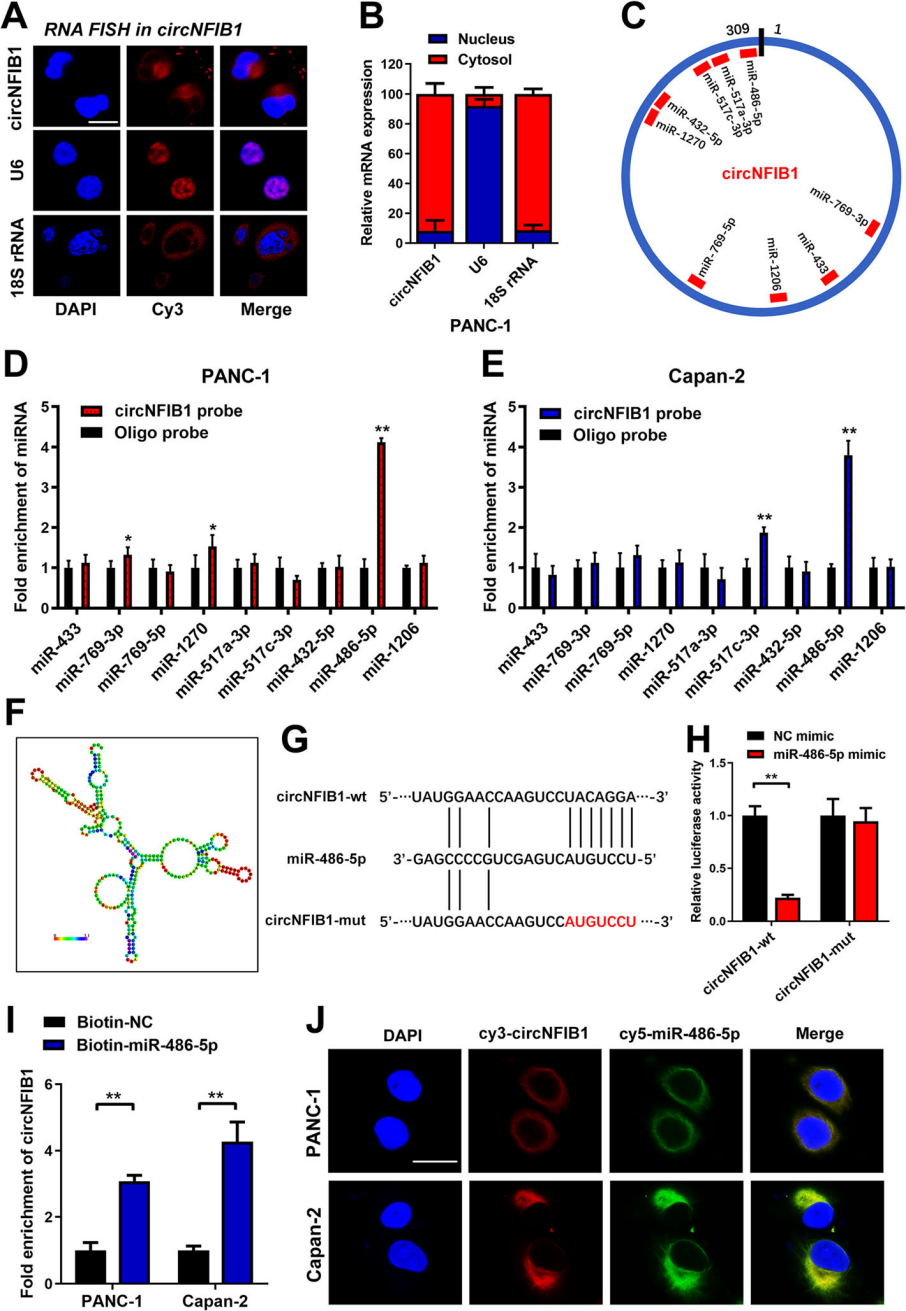
CircNFIB1 acts as a sponge for miR-486-5p in PDAC. **a and b** Representative FISH images (**a**) and subcellular fractionation assays (**b**) showed the subcellular distribution of circNFIB1 in Capan-2 cells. U6 was used as a nuclear control and 18S rRNA was used as a cytoplasmic control. Scale bar: 100 μm. **c** the potential target miRNAs of circNFIB1 were predicted using CircInteractome. **d and e** RNA pulldown assays revealed that miRNAs directly interact with circNFIB1 in PANC-1 (**d**) and Capan-2 (**e**) cells. **f** The secondary structure of circNFIB1 was predicted by RNAalifold. **g** Schematic illustrations showed the alignment of circNFIB1 with miR-486-5p and the red part indicates the mutagenesis nucleotides. **h** Dual luciferase reporter assays show the luciferase activity of wild-type or mutant circNFIB1 following co-transfection with either the miR-486-5p or control mimics. Relative firefly luciferase expression was normalized to that of Renilla luciferase. **i** RNA pulldown assays showed the RNAs captured by biotinylated miR-486-5p. **j** Representative FISH images showed the colocalization of circNFIB1 and miR-486-5p. Scale bar: 100 μm. Significance level was assessed using two-tailed Student *t*-tests and one-way ANOVA followed by Dunnett’s tests for multiple comparisons. Figures with error bars showed standard deviations of three independent experiments. *p < 0.05 and **p < 0.01

Since the localization of RNAs within cells are crucial for their functional display, we further determined the subcellular localization of circNFIB1 in PDAC cells. FISH and subcellular fractionation assays revealed that circNFIB1 was predominantly located in the cytoplasm (Fig. 5a, b). Cytoplasm-localized circRNAs primarily function as competitive endogenous RNAs (ceRNAs) and contribute to post-transcriptional regulations [12]. To determine whether circNFIB1 functions as an miRNA sponge in PDAC, nine miRNAs that potentially bound to circNFIB1 were predicted by CircInteractome [19] (Fig. 5c). Pull-down assays revealed that only miR-486-5p was enriched by circNFIB1 both in Capan-2 and PANC-1 cells (Fig. 5d, e). Moreover, luciferase reporter assays showed that miR-486-5p overexpression significantly decreased the luciferase activity of circNFIB1 compared with the NC group (Fig. 5f-h). After site-directed mutagenesis of the predicted complementary binding sites on circNFIB1, miR-486-5p failed to affect the luciferase activity of circNFIB1, which supported the sponge effect of circNFIB1 by binding to miR-486-5p on specific sequences (Fig. 5h). Furthermore, pull-down assays with biotin-labeled miR-486-5p and control showed that circNFIB1 was captured by miR-486-5p in PDAC cells, validating the interaction between circNFIB1 and miR-486-5p (Fig. 5i). Consistently, FISH assays confirmed the co-localization of circNFIB1 and miR-486-5p in the cytoplasm of PDAC cells (Fig. 5j).

**Fig. 6:**
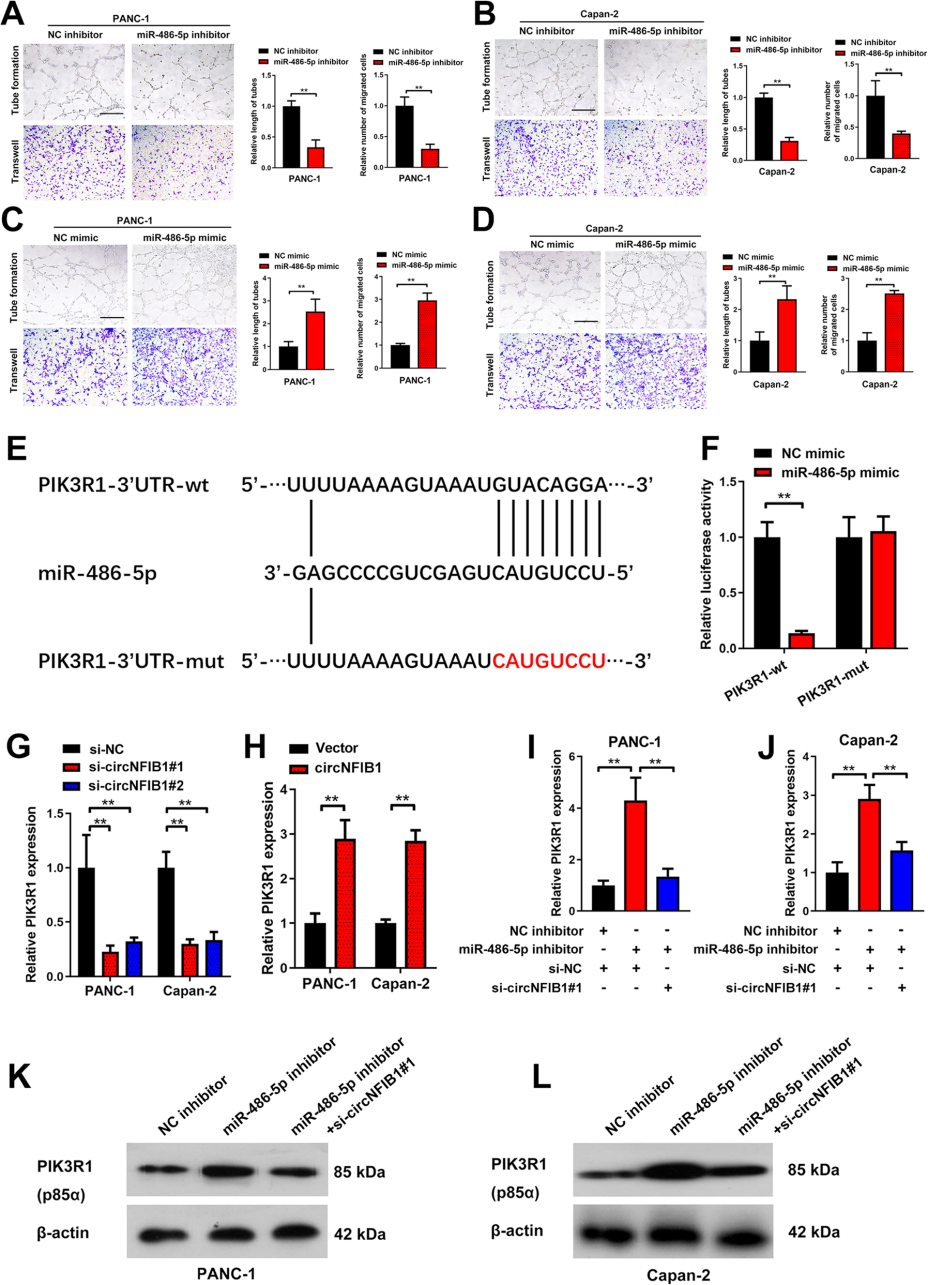
CircNFIB1 antagonizes miR-486-5p-mediated repression of PIK3R1 expression. **a-d** Representative images and a histogram analysis of tube formation and Transwell assay by HLECs treated with conditioned medium from miR-486-5p-silenced (**a and b**), miR-486-5p overexpressing (**c and d**), and corresponding control PDAC cells. Scale bar: 100 μm. **e** Schematic illustration showed the alignment of miR-486-5p with PIK3R1 and the red portion indicated the mutagenesis nucleotides. **f** Dual luciferase reporter assays showed the luciferase activity of wild type or mutant PIK3R1 following co-transfection with miR-486-5p mimic or control mimic. Relative firefly luciferase expression was normalized to that of Renilla luciferase. **g and h** The effect of circNFIB1-silencing or circNFIB1 overexpression on PIK3R1 expression in PDAC cells was assessed by qRT-PCR. **i and j** qRT-PCR analyzed the effect of circNFIB1-silencing on miR-486-5p depletion-induced PIK3R1 expression in PDAC cells. **k and l** Western blot analysis of circNFIB1-silencing on miR-486-5p depletion-induced PIK3R1 expression in PDAC cells. Significance level was assessed using two-tailed Student *t*-tests and one-way ANOVA followed by Dunnett’s tests for multiple comparisons. Figures with error bars showed the standard deviations of three independent experiments. *p < 0.05 and **p < 0.01

Since an interaction between circNFIB1 and miR-486-5p was determined, we further investigated whether miR-486-5p mediated lymphangiogenesis in PDAC. miR-486-5p knockdown suppressed PDAC cell-induced tube formation and the migration ability of HELCs (Fig. 6a, b), whereas miR-486-5p overexpression enhanced the ability of PDAC to induce HLEC tube formation and migration (Fig. 6c, d), suggesting that miR-486-5p promoted lymphangiogenesis in PDAC cells. Taken together, these results indicate that circNFIB1 directly binds to miR-486-5p and acts as an miR-486-5p sponge in PDAC cells.

### circNFIB1 antagonizes the miR-486-5p-mediated suppression of PIK3R1 expression

To identify the downstream targets regulated by miR-486-5p, the potential target genes of miR-486-5p were predicted by the intersection of TargetScan [20] and miRanda [21] (Additional file 4: Fig. S1d). Subsequently, the 95 predicted genes were subjected to pathway analysis using the PANTHER database [22] and 10 of the most enriched signaling pathways were recognized (Additional file 4: Fig. S1e). We further analyzed whether the predicted genes involved in these pathways were affected by miR-486-5p and found that PIK3R1 was negatively co-expressed with miR-486-5p in PDAC cells (Additional file 4: Fig. S1f-i). Moreover, miRNAs suppress target gene expression through binding to the 3’UTR of their mRNAs [23]. We found that the 3’UTR of PIK3R1 harbored sequences complementary to miR-486-5p sequences (Fig. 6e). The luciferase assays demonstrated that miR-486-5p reduced the luciferase activity of the PIK3R1 3’UTR luciferase-construct rather than the 3’UTR luciferase-construct with mutant sequences in the miR-486-5p binding site, indicating that miR-486-5p degraded PIK3R1 by directly targeting the 3’UTR region of PIK3R1 (Fig. 6f).

Next, we investigated whether circNFIB1 could induce PIK3R1 expression as a sponge of miR-486-5p. We found that circNFIB1 silencing significantly decreased the level of PI3KR1 expression in PDAC (Fig. 6g), whereas circNFIB1 overexpression increased the level of PIK3R1 (Fig. 6h), supporting the regulatory role of circNFIB1 on PIK3R1 expression. Importantly, miR-486-5p silencing upregulated PIK3R1 in PDAC, and downregulating circNFIB1 dramatically reversed this effect (Fig. 6i-l). Collectively, these findings suggest that circNFIB1 antagonizes the inhibitory effect of miR-486-5p on PIK3R1 expression.

### circNFIB1 inhibits LN metastasis via the miR-486-5p/PI3KR1/VEGF-C axis in PDAC

**Fig. 7:**
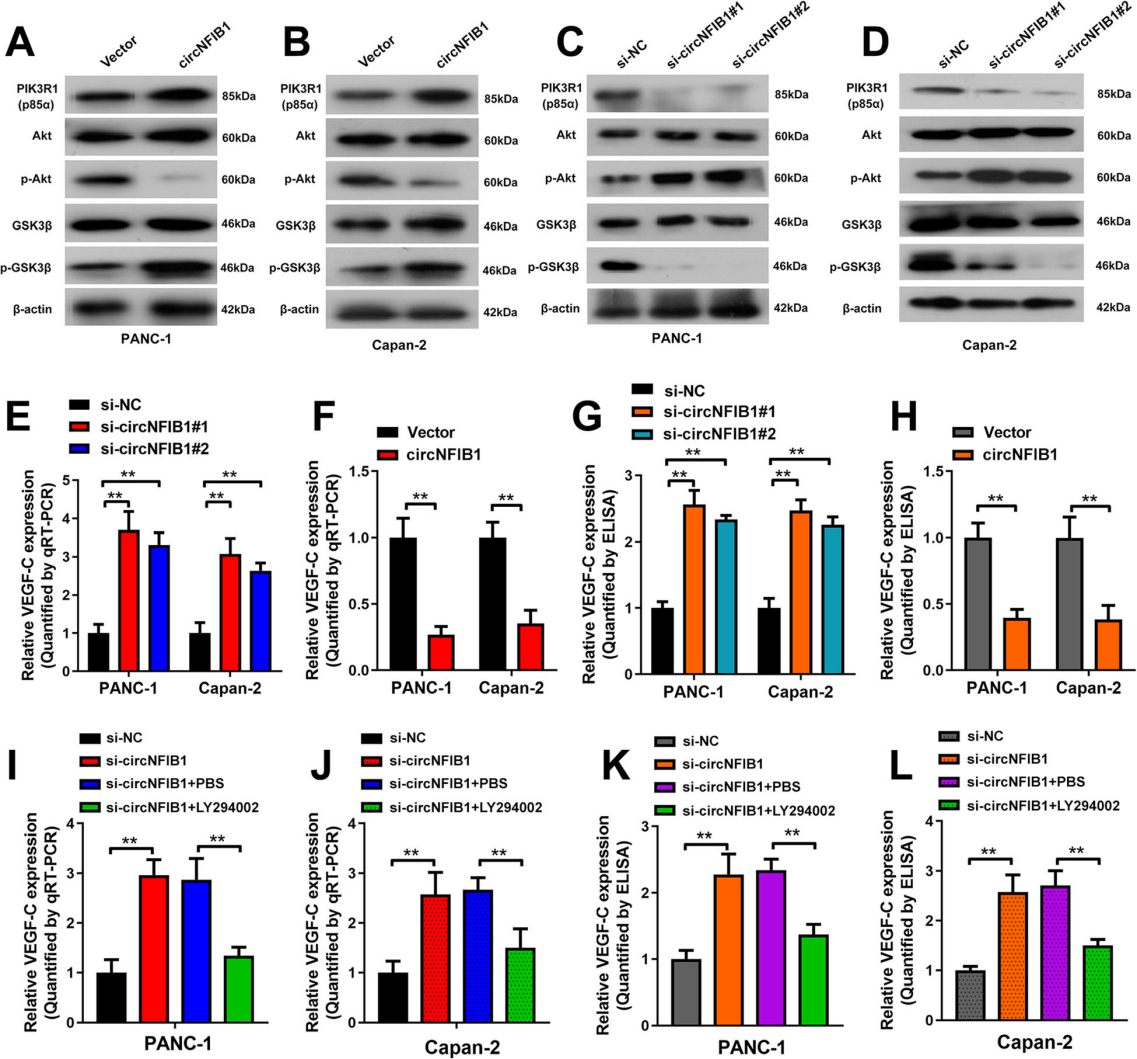
CircNFIB1 downregulates VEGF-C expression through inhibiting the PI3K/Akt signaling pathway. **a-d** Western blot analysis of PIK3R1, Akt, p-Akt, GSK3β, and p-GSK3β levels after the overexpression (**a and b**) or silencing (**c and d**) of circNFIB1 in PDAC cells. **e and f** The effect of circNFIB1-silencing (**e**) or circNFIB1-overexpression (**f**) on VEGF-C expression in PDAC was assessed by qRT-PCR. **g and h** ELISA analysis of VEGF-C secretion by PDAC after silencing (**g**) or overexpressing (**h**) circNFIB1. **i and j** The effect of LY294002 treatment on circNFIB1 depletion-induced VEGF-C expression in PDAC cells was assessed by qRT-PCR. **k and l** ELISA for LY294002 treatment on circNFIB1 depletion-induced VEGF-C secretion by PDAC cells. Significance level was assessed using two-tailed Student *t*-tests and one-way ANOVA followed by Dunnett’s tests for multiple comparisons. Figures with error bars showed the standard deviations of three independent experiments. *p < 0.05 and **p < 0.01

PIK3R1 (also known as PI3K p85α), a regulatory subunit of PI3K, has been reported to play an inhibitory role in the activation of the PI3K/Akt signaling pathway and suppress tumor progression [24, 25]. Thus, we explored whether circNFIB1 extensively regulates the activation of the PI3K/Akt signaling pathway in PDAC. The overexpression of circNFIB1 inhibited the phosphorylation of Akt and increased the level of PIK3R1 and phosphorylated GSK3β (Fig. 7a, b). Conversely, the downregulation of circNFIB1 expression facilitated the phosphorylation of Akt and reduced the level of PIK3R1 and phosphorylated GSK3β, suggesting that circNFIB1 upregulated PIK3R1 and inhibited activation of the PI3K/Akt signaling pathway in PDAC cells (Fig. 7c, d).

Since the role of circNFIB1 on the activation of the PI3K/Akt signaling pathway was clarified, the downstream targets of the PI3K/Akt signaling pathway associated with lymphangiogenesis in PDAC should be further determined. VEGF-C is required for lymphangiogenesis and considered to be a downstream regulator of the PI3K/Akt signaling pathway [26]. qRT-PCR analysis revealed that VEGF-C expression was increased by silencing circNFIB1 and decreased by ectopic circNFIB1 in PDAC cells (Fig. 7e, f). Consistently, Western blot and ELISA assay verified that the secretion of VEGF-C by PDAC cells was inhibited by circNFIB1 (Fig. 7g, h and Additional file 4: Fig. S1j-m). Moreover, we found that circNFIB1 depletion promoted VEGF-C expression and secretion; however, following treatment with LY294002, a PI3K/Akt signaling pathway inhibitor, the expression and secretion of VEGF-C were dramatically depleted (Fig. 7i-l), further confirming that circNFIB1 induced VEGF-C suppression via inactivation of the PI3K/Akt signaling pathway.

**Fig. 8:**
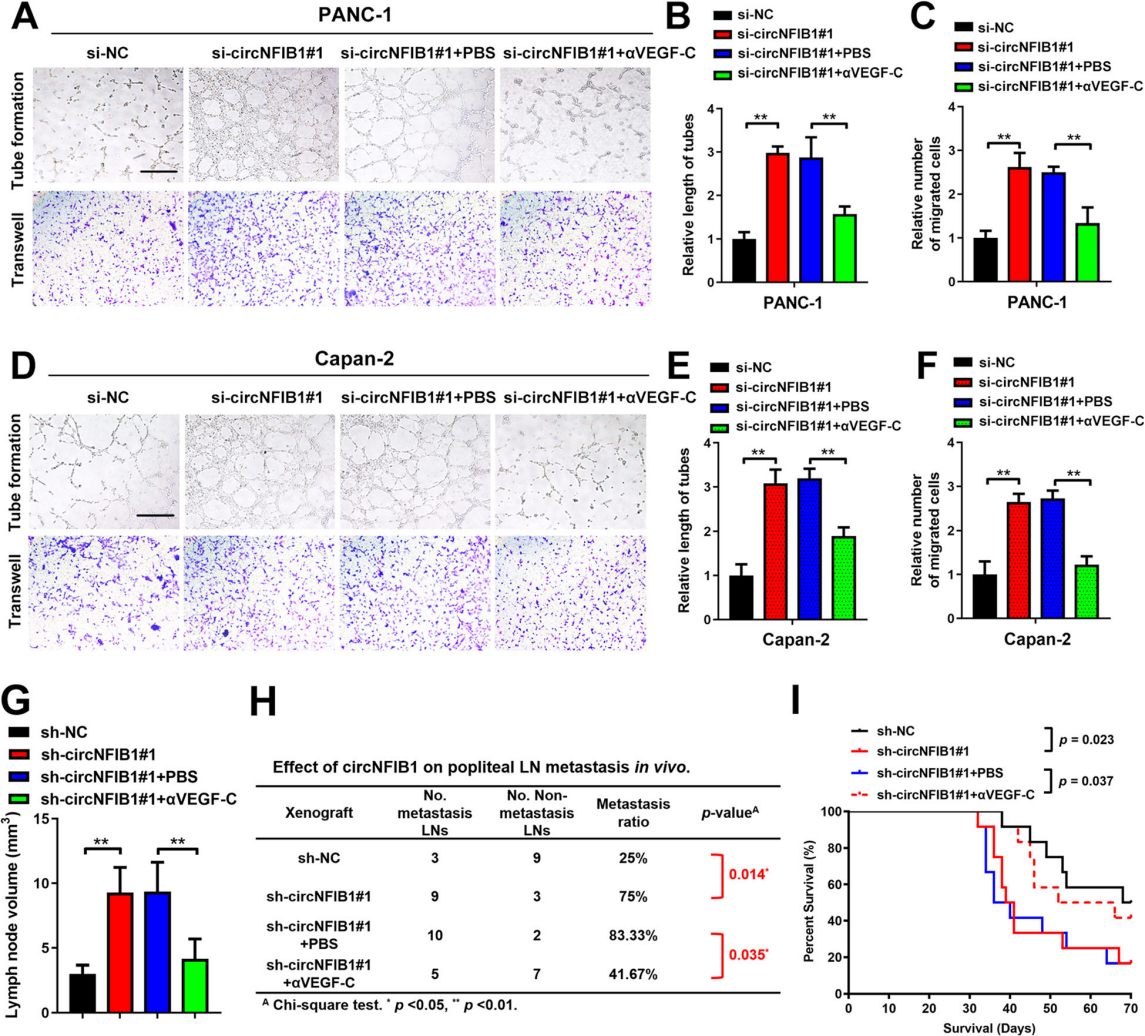
CircNFIB1 inhibits lymphangiogenesis and LN metastasis of PDAC via VEGF-C suppression. **a-f** Representative images (**a and d**) and a histogram analysis of tube formation (**b and e**) and Transwell (**c and f**) assay by HLECs treated with conditioned medium from si-NC, si-circNFIB1, si-circNFIB1 + PBS, and si-circNFIB1 + αVEGF-C PDAC cells. Scale bar: 100 μm. **g** Histogram analysis of the LN volume in the indicated groups. **h** The ratio of popliteal LN metastasis was calculated for all groups (*n* = 12 per group). **i** Kaplan-Meier survival curves for indicated groups (*n* = 12 per group). Significance level was assessed using two-tailed Student *t*-tests and one-way ANOVA followed by Dunnett’s tests for multiple comparisons. Figures with error bars showed the standard deviations of three independent experiments. *p < 0.05 and **p < 0.01

We next evaluated whether circNFIB1-mediated VEGF-C suppression contributed to the inhibition of lymphangiogenesis and LN metastasis in PDAC. Blocking VEGF-C with neutralizing antibody, pV1006R-r, dramatically reversed circNFIB1 depletion-induced HLEC tube formation and migration in PDAC (Fig. 8a-f). Consistently, in vivo assays showed that circNFIB1 knockdown increased tumor burden in the LNs and treatment with pV1006R-r inhibited circNFIB1-depletion-mediated LN metastasis (Fig. 8g). Moreover, administration of pV1006R-r decreased the LN metastatic rate of sh-circNFIB1#1 tumor bearing mice, which significantly prolonged the survival times (Fig. 8h, i). Together, these results reveal that circNFIB1 suppresses the LN metastasis of PDAC via the miR-486-5p/PI3K/VEGF-C axis.

### Clinical relevance of circNFIB1 as miR-486-5p sponge in PDAC patients

**Fig. 9:**
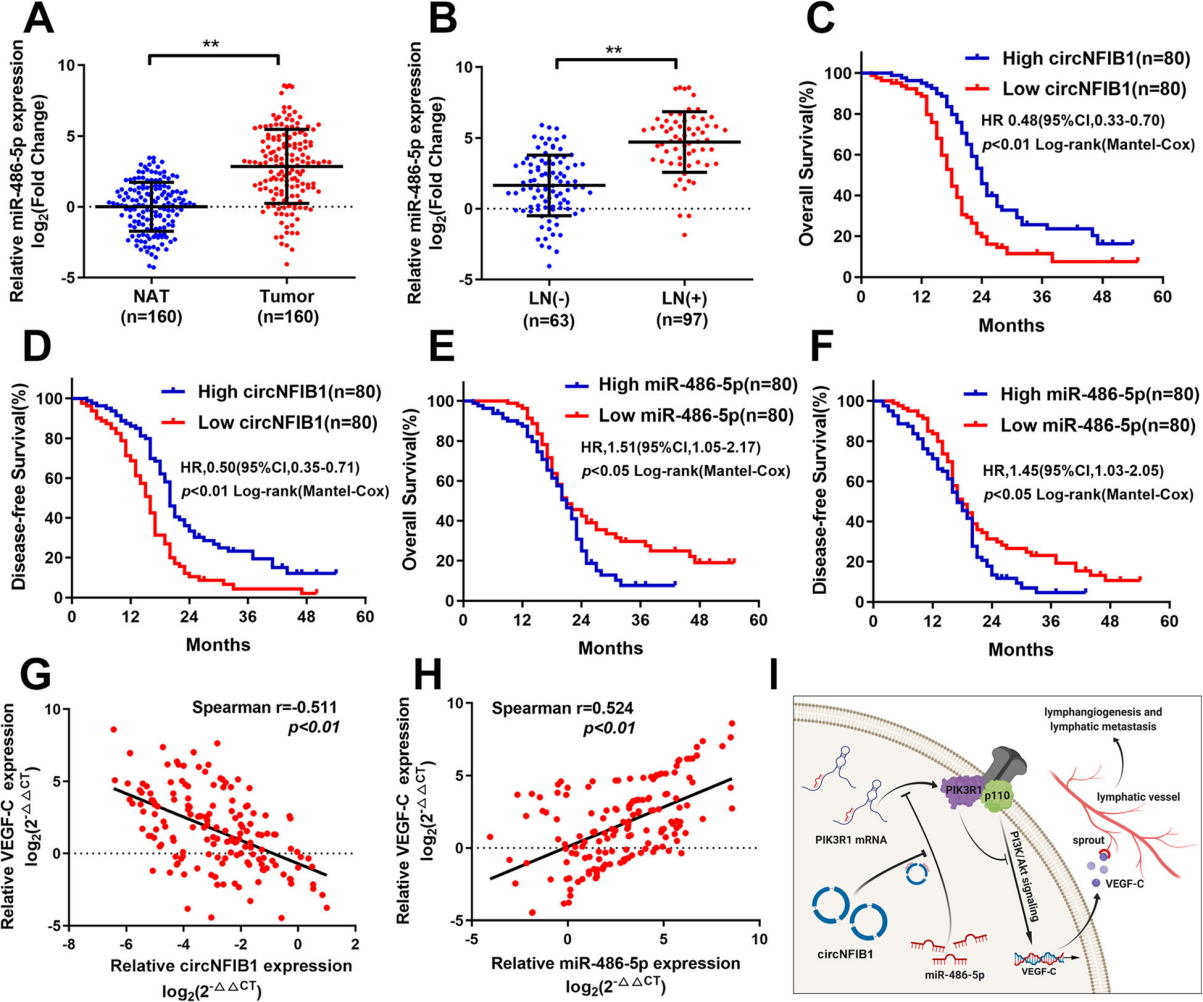
Clinical implication of circNFIB1 as miR-486-5p sponge in PDAC. **a** qRT-PCR analysis of miR-486-5p expression in PDAC tissues (*n* = 160) paired with NATs (*n* = 160). The nonparametric Mann-Whitney U test was used to compare different groups. **b** miR-486-5p expression in human PDAC tissues (*n* = 160) according to the LN status was assessed by qRT-PCR. The nonparametric Mann-Whitney U test was used to compare different groups. **c and d** Kaplan-Meier survival curves showed the OS (**c**) and DFS (**d**) of PDAC patients with low vs. high circNFIB1 expression. The cutoff value was the median expression of circNFIB1. **e and f** Kaplan-Meier survival curves showed the OS (**e**) and DFS (**f**) of PDAC patients with low vs. high miR-486-5p expression. The cutoff value was the median expression of miR-486-5p. **g** Correlation analysis of circNFIB1 and VEGF-C expression analyzed by qRT-PCR in PDAC tissues (*n* = 160). **h** Correlation analysis of miR-486-5p and VEGF-C expression analyzed by qRT-PCR in PDAC tissues (*n* = 160). **i** Proposed model for the role of circNFIB1 on promoting lymphangiogenesis and LN metastasis via the miR-486-5p/PIK3R1/VEGF-C axis in PDAC. *p < 0.05 and **p < 0.01

Given that the critical role of circNFIB1 in the inhibition of LN metastasis via sponging miR-486-5p was determined, we next assessed the clinical significance of circNFIB1 and miR-486-5p in a cohort of 160 PDAC patients. We found that miR-486-5p was overexpressed in PDAC tissues compared with NATs (Fig. 9a). Moreover, miR-486-5p expression in PDAC patients with LN metastasis was higher than those without LN metastasis (Fig. 9b). Importantly, Kaplan-Meier analysis revealed that high miR-486-5p expression or low circNFIB1 expression was associated with poor overall survival (OS) and disease-free survival (DFS) in 160 PDAC patients (Fig. 9c-f). Univariate and multivariate analyses demonstrated that circNFIB1 functioned as an independent factor for a good prognosis of PDAC patients (Additional file 5: Table S3 and Additional file 6: Table S4). Furthermore, correlation analysis demonstrated that VEGF-C levels were negatively correlated with circNFIB1 expression and positively associated with miR-486-5p expression in PDAC (Fig. 9g-h). In conclusion, our results indicate that circNFIB1 acts as a miR-486-5p sponge and mediates the inhibition of LN metastasis via VEGF-C suppression in PDAC patients.

## Discussion

CircRNAs represent a class of noncoding RNAs with a high degree of stability and participate in multiple biological processes and the regulation of gene expression in human cells [27]. However, little is known about the role and underlying mechanisms of circRNAs in the LN metastasis of PDAC. Herein, circNFIB1 was identified to be downregulated in PDAC and negatively correlated with LN metastasis in PDAC. Functionally, circNFIB1 suppressed lymphangiogenesis and LN metastasis in PDAC. Mechanistically, circNFIB1 functioned as a ceRNA and modulated the miR-486-5p/PI3KR1/VEGF-C axis, which inhibited the lymphangiogenesis and LN metastasis of PDAC. To our knowledge, this is the first report to provide insight into the regulatory mechanism of circRNA-mediated LN metastasis inhibition of PDAC and highlight that circNFIB1 may serve as a potential therapeutic target for LN metastasis in PDAC.

Lymphatic metastasis confers a poor prognosis for patients with cancer, including those with breast cancer and bladder cancer [28, 29]. Among the multiple factors associated with LN metastasis, lymphangiogenesis is considered to be essential for LN metastasis [30]. Yang et al. found that soluble VEGFR3 inhibited lymphangiogenesis, leading to further inhibition of lymphatic metastasis of bladder cancer [31]. Ji et al. showed that the TNFR1-mediated VEGF-C/VEGFR3 signaling pathway promoted lymphangiogenesis, resulting in LN metastasis [32]. However, few studies have focused on the regulation of lymphangiogenesis in PDAC. In this study, we demonstrated that circNFIB1 overexpression was negatively correlated with the lymphatic vessel density in a 160-case cohort of PDAC patients. Furthermore, circNFIB1 inhibited lymphangiogenesis in PDAC through reducing the expression of VEGF-C and ultimately limited LN metastasis in PDAC. Thus, our findings clarified the molecular mechanism underlying circNFIB1 overexpression and the inhibition LN metastasis, which provides novel insight into the regulatory mechanism of LN metastasis in PDAC.

VEGF-C is a VEGFR3 receptor ligand, which is expressed on lymphatic vascular endothelial cells and is critical for lymphangiogenesis [11]. The disfunction of VEGF-C blocks the development of lymphatic vessels and inhibits lymphatic metastasis of cancer cells [33]. Chen et al. found that LNMAT1 promoted the expression of CCL2 and recruited macrophages to increase VEGF-C and facilitate the lymphangiogenesis of bladder cancer [34]. Similarly, Liu et al. demonstrated that TBL1XR1 bound to the VEGF-C promotor to induce VEGF-C expression and promoted lymphangiogenesis and LN metastasis in esophageal squamous cell carcinoma [35]. Although the importance of VEGF-C in lymphangiogenesis and LN metastasis has been determined in multiple cancers, the mechanism underlying the regulation of VEGF-C in PDAC remains unclear. In the present study, we reported that circNFIB1 downregulated VEGF-C expression through directly sponging miR-486-5p and inactivating the PI3K/AKT signaling pathway, which further inhibited lymphangiogenesis and LN metastasis of PDAC. Therefore, our study revealed a molecular mechanism for circRNA-induced VEGF-C suppression and indicated that circNFIB1 may be a potential target by which to block VEGF-C-induced LN metastasis in PDAC patients.

The PI3K/Akt signaling pathway has been thoroughly demonstrated to be a key regulator in cancer progression. Overactivation of this pathway has been observed in a variety of cancers, including PDAC [36]. Moreover, it has been reported that activation of the PI3K/Akt signaling pathway contributed to LN metastasis in several cancers. Yoo et al. revealed that Shh signaling promoted the lymphatic metastasis of gastric cancer through activation of the PI3K/Akt pathway [37]. In addition, Saal et al. reported that a PIK3CA mutation induced the activation of the PI3K/Akt signaling pathway and facilitated LN metastasis in breast cancer [38]. Nevertheless, little has been described about the PI3K/Akt pathway and LN metastasis of PDAC. In the present study, we found that circNFIB1 induced the upregulation of PIK3R1, and the regulatory subunit of PI3K could inhibit activation of the PI3K/Akt signaling pathway and reduce VEGFC expression, thereby limiting lymphangiogenesis and LN metastasis of PDAC. Moreover, blocking the PI3K/Akt signaling pathway with its inhibitor, LY294002, significantly decreased circNFIB1 depletion-induced VEGF-C, which serves as the key regulator for lymphangiogenesis. Thus, inhibition of PI3K/Akt signaling pathway may provide a novel intervention strategy for LN metastasis in PDAC.

## Conclusion

In summary, we uncovered a novel mechanism by which circRNA-induced VEGF-C inhibition suppressed LN metastasis of PDAC. We clinically demonstrated that the downregulation of circNFIB1 is associated with the poor prognosis of PDAC patients. We further indicated that circNFIB1 overexpression could effectively inhibit LN metastasis via the miR-486-5p/PIK3R1/VEGF-C axis. Identification of circNFIB1, which inhibits LN metastasis, both expands our knowledge of LN metastasis regulation and develops a potential therapeutic target for LN metastasis in PDAC.

## Supplementary information

**Additional file 1 Table S1.** Correlation between circNFIB1 expression and clinicopathologic characteristics of PDAC patients

**Additional file 2 Table S2.** Primers and probes used in the experiments.

**Additional file 3.** Supplementary methods.

**Additional file 4 Figure S1.** The identification of the downstream targets of miR-486-5p.

**Additional file 5 Table S3.** Univariate and multivariate analyses of OS for circNFIB1 expression in PDAC patients.

**Additional file 6 Table S4.** Univariate and multivariate analyses of DFS for circNFIB1 expression in PDAC patients.

**Additional file 7 Table S5.** Antibodies used in the experiments.

**Additional file 8 Figure S2.** Full uncut original pictures.

**Additional file 9 Figure S3.** Full uncut original gels.

## Data Availability

Our circRNAs next-generation sequencing data used in this study have been deposited in the NCBI’s Gene Expression Omnibus and are accessible through GEO accession number: GSE136569 (https://www.ncbi.nlm.nih.gov/geo/query/acc.cgi?acc=GSE136569).

## Abbreviations

PDAC: Pancreatic ductal adenocarcinoma
circRNAs: Circular RNAs
LN: Lymph node
VEGF-C: Vascular endothelial growth factor-C
NATs: Normal adjacent tissues
qRT-PCR: Quantitative real-time PCR
OS: Overall survival
DFS: Disease-free survival
HPDE: Human pancreatic ductal endothelial cells
FISH: Fluorescence in situ hybridization
IHC: Immunohistochemistry
HLECs: Human lymphatic endothelial cells

## References

[1] Ellis C, Ramzy A, Kieffer TJ (2017). Regenerative medicine and cell-based approaches to restore pancreatic function. Nat Rev Gastroenterol Hepatol.

[2] Makohon-Moore A, Iacobuzio-Donahue CA (2016). Pancreatic cancer biology and genetics from an evolutionary perspective. Nat Rev Cancer.

[3] Vincent A, Herman J, Schulick R, Hruban RH, Goggins M (2011). Pancreatic cancer. Lancet.

[4] Philip PA, Mooney M, Jaffe D, Eckhardt G, Moore M, Meropol N, Emens L, O'Reilly E, Korc M, Ellis L (2009). Consensus report of the national cancer institute clinical trials planning meeting on pancreas cancer treatment. J Clin Oncol.

[5] Groot VP, Gemenetzis G, Blair AB, Rivero-Soto RJ, Yu J, Javed AA, Burkhart RA, Rinkes I, Molenaar IQ, Cameron JL (2019). Defining and predicting early recurrence in 957 patients with resected pancreatic ductal adenocarcinoma. Ann Surg.

[6] Paniccia A, Hosokawa P, Henderson W, Schulick RD, Edil BH, McCarter MD, Gajdos C (2015). Characteristics of 10-year survivors of pancreatic ductal adenocarcinoma. JAMA Surg.

[7] Sundar SS, Ganesan TS (2007). Role of lymphangiogenesis in cancer. J Clin Oncol.

[8] Achen MG, McColl BK, Stacker SA (2005). Focus on lymphangiogenesis in tumor metastasis. Cancer Cell.

[9] Cao Y (2005). Opinion: emerging mechanisms of tumour lymphangiogenesis and lymphatic metastasis. Nat Rev Cancer.

[10] Stacker SA, Williams SP, Karnezis T, Shayan R, Fox SB, Achen MG (2014). Lymphangiogenesis and lymphatic vessel remodelling in cancer. Nat Rev Cancer.

[11] Jussila L, Alitalo K (2002). Vascular growth factors and lymphangiogenesis. Physiol Rev.

[12] Kristensen LS, Andersen MS, Stagsted LVW, Ebbesen KK, Hansen TB, Kjems J (2019). The biogenesis, biology and characterization of circular RNAs. Nat Rev Genet.

[13] Beermann J, Piccoli MT, Viereck J, Thum T (2016). Non-coding RNAs in development and disease: background, mechanisms, and therapeutic approaches. Physiol Rev.

[14] Yang C, Yuan W, Yang X, Li P, Wang J, Han J, Tao J, Li P, Yang H, Lv Q, Zhang W (2018). Circular RNA circ-ITCH inhibits bladder cancer progression by sponging miR-17/miR-224 and regulating p21, PTEN expression. Mol Cancer.

[15] Wei Y, Chen X, Liang C, Ling Y, Yang X, Ye X, Zhang H, Yang P, Cui X, Ren Y (2019). A noncoding regulatory RNAs network driven by Circ-CDYL acts specifically in the early stages hepatocellular carcinoma. Hepatology.

[16] Rupaimoole R, Slack FJ (2017). MicroRNA therapeutics: towards a new era for the management of cancer and other diseases. Nat Rev Drug Discov.

[17] Lopez-Bertoni H, Kotchetkov IS, Mihelson N, Lal B, Rui Y, Ames H, Lugo-Fagundo M, Guerrero-Cazares H, Quinones-Hinojosa A, Green JJ, Laterra J. A Sox2/miR-486-5p axis regulates survival of GBM cells by inhibiting tumor suppressor networks. Cancer Res. 2020;80:1644–55. 10.1158/0008-5472.CAN-19-1624 PMC7165043 32094299

[18] Hanna JA, Garcia MR, Lardennois A, Leavey PJ, Maglic D, Fagnan A, Go JC, Roach J, Wang YD, Finkelstein D, Hatley ME (2018). PAX3-FOXO1 drives miR-486-5p and represses miR-221 contributing to pathogenesis of alveolar rhabdomyosarcoma. Oncogene.

[19] Dudekula DB, Panda AC, Grammatikakis I, De S, Abdelmohsen K, Gorospe M. CircInteractome: A web tool for exploring circular RNAs and their interacting proteins and microRNAs. RNA Biol. 2016;13:34–42. 10.1080/15476286.2015.1128065 PMC4829301 26669964

[20] Agarwal V, Bell GW, Nam JW, Bartel DP. Predicting effective microRNA target sites in mammalian mRNAs. Elife. 2015;4:e05005. 10.7554/eLife.05005 PMC4532895 26267216

[21] Betel D, Wilson M, Gabow A, Marks DS, Sander C. The microRNA.org resource: targets and expression. Nucleic Acids Res. 2008;36:D149–53. 10.1093/nar/gkm995 PMC2238905 18158296

[22] Mi H, Huang X, Muruganujan A, Tang H, Mills C, Kang D, Thomas PD. PANTHER version 11: expanded annotation data from Gene Ontology and Reactome pathways, and data analysis tool enhancements. Nucleic Acids Res. 2017;45:D183–9. 10.1093/nar/gkw1138 PMC5210595 27899595

[23] Mori MA, Ludwig RG, Garcia-Martin R, Brandao BB, Kahn CR (2019). Extracellular miRNAs: from biomarkers to mediators of physiology and disease. Cell Metab.

[24] Taniguchi CM, Winnay J, Kondo T, Bronson RT, Guimaraes AR, Aleman JO, Luo J, Stephanopoulos G, Weissleder R, Cantley LC, Kahn CR (2010). The phosphoinositide 3-kinase regulatory subunit p85alpha can exert tumor suppressor properties through negative regulation of growth factor signaling. Cancer Res.

[25] Luo J, Sobkiw CL, Logsdon NM, Watt JM, Signoretti S, O'Connell F, Shin E, Shim Y, Pao L, Neel BG (2005). Modulation of epithelial neoplasia and lymphoid hyperplasia in PTEN+/− mice by the p85 regulatory subunits of phosphoinositide 3-kinase. Proc Natl Acad Sci U S A.

[26] Brouillard P, Boon L, Vikkula M (2014). Genetics of lymphatic anomalies. J Clin Invest.

[27] Patop IL, Wust S, Kadener S (2019). Past, present, and future of circRNAs. EMBO J.

[28] McAllaster JD, Cohen MS (2011). Role of the lymphatics in cancer metastasis and chemotherapy applications. Adv Drug Deliv Rev.

[29] Chen C, Luo Y, He W, Zhao Y, Kong Y, Liu H, Zhong G, Li Y, Li J, Huang J (2020). Exosomal long noncoding RNA LNMAT2 promotes lymphatic metastasis in bladder cancer. J Clin Invest.

[30] Zheng W, Aspelund A, Alitalo K (2014). Lymphangiogenic factors, mechanisms, and applications. J Clin Invest.

[31] Yang H, Kim C, Kim MJ, Schwendener RA, Alitalo K, Heston W, Kim I, Kim WJ, Koh GY (2011). Soluble vascular endothelial growth factor receptor-3 suppresses lymphangiogenesis and lymphatic metastasis in bladder cancer. Mol Cancer.

[32] Ji H, Cao R, Yang Y, Zhang Y, Iwamoto H, Lim S, Nakamura M, Andersson P, Wang J, Sun Y (2014). TNFR1 mediates TNF-alpha-induced tumour lymphangiogenesis and metastasis by modulating VEGF-C-VEGFR3 signalling. Nat Commun.

[33] Karaman S, Detmar M (2014). Mechanisms of lymphatic metastasis. J Clin Invest.

[34] Chen C, He W, Huang J, Wang B, Li H, Cai Q, Su F, Bi J, Liu H, Zhang B (2018). LNMAT1 promotes lymphatic metastasis of bladder cancer via CCL2 dependent macrophage recruitment. Nat Commun.

[35] Liu L, Lin C, Liang W, Wu S, Liu A, Wu J, Zhang X, Ren P, Li M, Song L (2015). TBL1XR1 promotes lymphangiogenesis and lymphatic metastasis in esophageal squamous cell carcinoma. Gut.

[36] LoRusso PM (2016). Inhibition of the PI3K/AKT/mTOR pathway in solid tumors. J Clin Oncol.

[37] Yoo YA, Kang MH, Lee HJ, Kim BH, Park JK, Kim HK, Kim JS, Oh SC (2011). Sonic hedgehog pathway promotes metastasis and lymphangiogenesis via activation of Akt, EMT, and MMP-9 pathway in gastric cancer. Cancer Res.

[38] Saal LH, Holm K, Maurer M, Memeo L, Su T, Wang X, Yu JS, Malmstrom PO, Mansukhani M, Enoksson J (2005). PIK3CA mutations correlate with hormone receptors, node metastasis, and ERBB2, and are mutually exclusive with PTEN loss in human breast carcinoma. Cancer Res.

