# Supplementary material for: circNFIB1 inhibits lymphangiogenesis and lymphatic metastasis via the miR-486-5p/PIK3R1/VEGF-C axis in pancreatic cancer
Source: Mol Cancer. 2020 May 4;19:82. doi: 10.1186/s12943-020-01205-6 (PMC7197141; doi:10.1186/s12943-020-01205-6)
Supplement: Supplementary file 1 — Additional file 1 Table S1. Correlation between circNFIB1 expression and clinicopathologic characteristics of PDAC patients [file 12943_2020_1205_MOESM1_ESM.doc]

**Table S1. Correlation between circNFIB1 expression and clinicopathologic characteristics of PDAC patients**

| **Characteristics** | **No. of cases** | **circNFIB1 expression level** | | |
| --- | --- | --- | --- | --- |
|  |  | **Low** | **High** | ***p*-value^a^** |
| **Total cases** | 160 | 80 | 80 |  |
| **Gender** |  |  |  | 0.262 |
| Male | 93 | 43 | 50 |  |
| Female | 67 | 37 | 30 |  |
| **Age** |  |  |  | 0.327 |
| ≤60 | 60 | 27 | 33 |  |
| ＞60 | 100 | 53 | 47 |  |
| **Differentiation** |  |  |  | 0.260 |
| Poor | 30 | 13 | 17 |  |
| Moderate | 98 | 47 | 51 |  |
| Well | 32 | 20 | 12 |  |
| **T stage** |  |  |  | 0.426 |
| T1-2 | 89 | 42 | 47 |  |
| T3-4 | 71 | 38 | 33 |  |
| **Lymphatic metastasis** |  |  |  | **0.001^**^** |
| Negative | 63 | 11 | 52 |  |
| Positive | 97 | 69 | 28 |  |
| **TNM stage** |  |  |  | **0.001^**^** |
| Stage I | 34 | 5 | 29 |  |
| Stage II | 82 | 43 | 39 |  |
| Stage III | 44 | 32 | 12 |  |

Abbreviations: No. of cases = number of cases; T stage = tumor stage; TNM stage = tumor node metastasis stage. ^a^ Chi-square test, ^*^ *p* <0.05, ^**^ *p* <0.01.
