# Supplementary material for: circNFIB1 inhibits lymphangiogenesis and lymphatic metastasis via the miR-486-5p/PIK3R1/VEGF-C axis in pancreatic cancer
Source: Mol Cancer. 2020 May 4;19:82. doi: 10.1186/s12943-020-01205-6 (PMC7197141; doi:10.1186/s12943-020-01205-6)
Supplement: Supplementary file 2 — Additional file 2 Table S2. Primers and probes used in the experiments. [file 12943_2020_1205_MOESM2_ESM.doc]

**Table S2. Primer and probes used in the experiments.**

| **Gene** | **Sequence (5’-3’)** | **Application** |
| --- | --- | --- |
| NFIB | F: CCTGCAACGTAGCCTTTGTT  R: CCTAGCCTTCGTTGGTGAGA | qRT-PCR |
| PIK3R1 | F: TGGTATTATGGGTGGGGAGG  R: GCATCCTGGGCAGCTAATG | qRT-PCR |
| circNFIB1 | F: TACCCCTCTCCAAGTTCACC  R: GGTTATGGGCGTTCTGGATA | qRT-PCR |
| miR-1270 | F: CTGGAGATATGGAAGAGCTG | qRT-PCR |
| miR-432-5p | F: TCTTGGAGTAGGTCATTGGG | qRT-PCR |
| miR-517c-3p | F: ATCGTGCATCCTTTTAGAGTG | qRT-PCR |
| miR-517a-3p | F: ATCGTGCATCCCTTTAGAGT | qRT-PCR |
| miR-486-5p | F: TCCTGTACTGAGCTGCCCC | qRT-PCR |
| miR-769-3p | F: CTGGGATCTCCGGGGTCTTGGTT | qRT-PCR |
| miR-433 | F: ATCATGATGGGCTCCTCGGTGT | qRT-PCR |
| miR-1206 | F: TGTTCATGTAGATGTTTAAGC | qRT-PCR |
| miR-769-5p | F: TGAGACCTCTGGGTTCTGAGCT | qRT-PCR |
| U6 | F: CTCGCTTCGGCAGCACA  R: AACGCTTCACGAATTTGCGT | qRT-PCR |
| 18S rRNA | F: ACACGGACAGGATTGACAGA  R: GGACATCTAAGGGCATCACA | qRT-PCR |
| GAPDH | F: CATGAGAAGTATGACAACAGCCT  R: AGTCCTTCCACGATACCAAAGT | qRT-PCR |
| circNFIB1 probe | AACUAUCCUCAAGGUAACCUUGAUCUCUU | RNA pulldown |
| si-circNFIB1#1 | sense: CGAAAGAGAUCAAGGUUACTT  antisense: GUAACCUUGAUCUCUUUCGTT | si-RNA |
| si-circNFIB1#2 | sense: GAGAUCAAGGUUACCUUGATT  antisense: UCAAGGUAACCUUGAUCUCTT | si-RNA |
| NC mimics | sense: UUCUCCGAACGUGUCACGUTT  antisense: ACGUGACACGUUCGGAGAATT | miRNA mimics |
| miR-486-5p mimics | sense: UCCUGUACUGAGCUGCCCCGAG  antisense: CUCGGGGCAGCUCAGUACAGGA | miRNA mimics |
| NC inhibitor | CAGUACUUUUGUGUAGUACAA | miRNA inhibitor |
| miR-486-5p inhibitor | CUCGGGGCAGCUCAGUACAAGGA | miRNA inhibitor |
