# Supplementary material for: circNFIB1 inhibits lymphangiogenesis and lymphatic metastasis via the miR-486-5p/PIK3R1/VEGF-C axis in pancreatic cancer
Source: Mol Cancer. 2020 May 4;19:82. doi: 10.1186/s12943-020-01205-6 (PMC7197141; doi:10.1186/s12943-020-01205-6)
Supplement: Supplementary file 3 — Additional file 3. Supplementary methods. [file 12943_2020_1205_MOESM3_ESM.doc]

**Supplemental methods**

*Lentivirus infection and Cell transfection*

For Lentivirus infection, PDAC cells were seeded in 6-well plates and transfected with lentiviral vector which could transfer circNFIB1 shRNA in PDAC cells, this process was performed using X-tremeGENE HP reagent (Roche). Lentiviral vector was obtained by transfection of 293T cells.

For cellular transfection, PDAC cells and HLECs were pre-seeded in 6-well plates the day before transfection, then circNFIB1 siRNAs (GenePharma, Shanghai, China), miRNA mimics, miRNA inhibitor (Sangon Biotech, Shanghai, China) were transfected into these cells with lipofectamine 3000 or P3000 (Life Technologies, CA, USA) according to the manufacture’s introduction. After 48 hours, the efficiency of cells transfection was confirmed via qRT-PCR or Western blotting analysis. All the sequences used in this study are listed in Additional file 2.

***RNA extraction and quantitative real-time PCR (qRT-PCR)***

Total RNAs were extracted from patient specimens or PDAC cells using TRIzol reagent (Invitrogen Life Technologies) following the manufactory’s instructions. Then total RNA was transcribed into cDNA using PrimerScript RT Master Mix (Takara, Japan). qRT-PCR was performed using TB Green Premix Ex Taq II (Takara, Japan). The process was conducted on Light Cycler 480 Detection System (Roche), and the results were normalized to the expression of GAPDH. For miRNA, cDNA and qRT-PCR were performed with Mir-XTM miRNA First-Strand Synthesis Kit (Takara, Japan). All the primers were listed in Additional file 2.

***HLECs tube formation assay and Transwell assay***

PDAC cells were seeded in 6-well plates and treated with circNFIB1 siRNA or plasmids, 48 hours later, the cell culture media was collected and concentrated 10 folds using ultrafiltration spin columns (Millipore, Billerica, MA, USA).

For tube formation assay, HLECs were seeded into 12-well plates which were pre-coated with Matrigel. Then concentrated cell culture media was added to the plate. 12 hours later, the lymphatic tubes were imaged with an inverted microscope and the number and area of completed tubule structures were measured and quantified.

For transwell assay, HLECs were incubated with concentrated cell culture media for 48 hours. Then 2×10^5^ HLECs suspended with serum-free medium were seeded to the upper chamber of Matrigel-coated Transwell plates. 700ul complete culture media was applied to the lower chamber of Transwell chambers (BD Biosciences, MA, USA). 24 hours’ later, the cells remaining in the upper chamber were scrapped off and the invaded cells were fixed with 4% paraformaldehyde, stained crystal violet, and the number of invading cells were calculated in 5 random fields.

***Subcellular fractionation assay***

The cytoplasmic and nuclear fraction of PDAC cells were separated using the NE-PER™ Nuclear and Cytoplasmic Extraction Reagents, according to the manufactory’s instruction. Briefly, 1 x 10^7^ PDAC cells were harvested and re-suspended with PBS. Then CER I, CERII were added into the suspension. After incubating on ice for 10 min, the suspension was centrifuged at 16000 x g for 5 min and the supernatant with plasma portion was kept. The pellet was resuspended with ice-cold NER and vortexed 15 seconds every 10 min for four times. Then the suspension was centrifuged for 10 min at 16,000 x g and the supernatant with nuclear fraction was collected. The plasma portion and nuclear fraction was kept at -80°C until use.

***Fluorescence in situ hybridization (FISH)***

The cells were seeded in confocal dished and fixed with 4% [paraformaldehyde](javascript:;) when reached 80–90% confluence and permeabilizated with 0.5% Triton. After washing with PBS for three times, the cells were hybridized with Cy3-labeled circNFIB1 probe (Sangon biotech, Shanghai) at 37 °C overnight, cell nuclei were stained with DAPI. The images were capture with Zeiss LAM 710 focal Microscope (Carl Zeiss AG, Germany).

***Colocalization of circNFIB1 and miR-486a-5p***

The colocalization of circNFIB1 and miR-486a-5p was confirmed by fluorescence in situ hybridization. Briefly, PANC-1 and Capan-2 cells were seeded on a confocal plate and when reached 80-90% confluence, the cells were fixed with 4% [paraformaldehyde](javascript:;) and permeabilizated with 0.5% Triton. After washing with PBS for three times, the cells were hybridized with Cy3-labeled circNFIB1 probe and Cy5-labeled miR-486-5p probe (GenePharma, China) overnight at 37°C in 2×SSC, 10% formamide and 10% dextran. Cell nuclei were stained with DAPI. The images were capture with Zeiss LAM 710 focal Microscope (Carl Zeiss AG, Germany).

***Luciferase activity assay***

1×10^5^ PDAC cells were seeded in 6-well plates one day before transfection. Then plasmids carrying wild-type circNFIB1 or wild-type PIK3R1 3’UTR sequences were co-transfected with miR-486-5p mimics or NC mimic into the pre-seeded cells; mutant sequences of circNFIB1 or PIK3R1 3’UTR were also co-transfected with miR-1246 mimics or NC mimics into the pre-seeded cells. Then the transfected Cells were seeded into 96-well plates at a density of 1×10^4^ cells per well, the luciferase activities was measured according to the instruction of Dual-Luciferase Reporter Assay (Promega, USA).

***Western blotting***

To determine the protein levels in cells, cells were washed with PBS for three times and treated with RIPA buffer (Invitrogen) mixed with 1% Protease Inhibitor Cocktail and 1% phosphatase inhibitor. Then the lysates were centrifuged at 12000 rpm for 30 min, the supernatants were collected and the protein concentration were calculated with bicinchoninic acid protein assay kit (Pierce, Rockford, IL, USA). For Western blot analysis of VEGF-C, samples were obtained from total culture media that was 100-fold concentrated using Amicon Ultra-4 columns (Millipore, Billerica, MA, USA). Later, concentrated culture media or 30ug of proteins from cell lysate were subjected to SDS‐polyacrylamide gel electrophoresis and transferred to a polyvinylidene fluoride membrane. After incubation with 5% BSA, the blots were treated with primary antibodies overnight at 4 ℃, and washed with TBST three times before incubated with secondary antibodies. ECL chemiluminescence kit (Pierce) were used to detect bound antibody. All the antibodies used in this study are listed in Additional file 7. Full uncut original pictures were shown in Additional file 8.

***Immunohistochemistry***

Paraffin-embedded samples were deparaffinized in xylene twice, and rehydrated in [absolute](javascript:;) [ethyl](javascript:;) [alcohol](javascript:;) twice, followed by grade series of ethanol. Then the samples were submerged in 10 mmol/l citrate buffer (pH = 6.0), and heated in a microwave oven for 21 min for epitope retrieval. The activity of endogenous peroxidase was blocked with 3% hydrogen peroxide at room temperature, for 10 min. Then the samples were blocked in goat serum for 20 min and incubated with rabbit anti-human LYVE-1 antibody overnight at 4°C. After washing with PBS for three times, the samples were stained with corresponding secondary antibody for 30 min, at room temperature and counterstained with hematoxylin. The immunohistochemistry analyses were scored by 2 independent professional pathologists. Briefly, the proportion of positive staining tumor cells was graded as follows: 0, no positive tumor cells; 1, 0%–10% positive tumor cells; 2, 10%–30% positive tumor cells; 3, 30%–70% positive tumor cells; and 4, more than 70% positive tumor cells. The staining intensity was graded as follows: 1, no staining; 2, weak staining (light yellow); 3, moderate staining (yellow brown for IHC); and 4, strong staining (brown for IHC). The staining index (SI) was calculated by multiplying the staining intensity and the proportion of positive tumor cells, with possible scores of 0, 1, 2, 3, 4, 6, 8, 9, 12, and 16. Images were visualized using a Nikon ECLIPSE Ti (Fukasawa, Japan) microscope system and processed with Nikon software.

***ELISA-based quantification of secreted VEGF-C.***

ELISA assay was performed according to the instruction of Human VEGF-C Quantikine ELISA Kit (Cat. No. DVEC00, 17 R&D). Briefly, add 75ul/well VEGF-C antigen to coat the plate, and incubated the plate at 4 °C overnight. After washing the plate with ELISA buffer three times, blocking the plate with blocking buffer and incubating at normal temperature for 60 min. Washing the plate three times and add 75ul/well antibody conjugate solution, then incubating the plate at room temperature for 1 hour. Remove the solution and wash the plate 6 times with wash buffer in an ELISA washer. Add 75ul/well substrate solution to the plate and incubated it at room temperature for 30 min until reached the desired color intensity. Add 75ul/well stop solution to stop the reaction and measure the absorbance with [spectrophotometer](javascript:;)  Read the light signal (relative light units) using PerkinElmer VICTOR™ X3 Multilabel Plate Reader.

***Gel Electrophoresis Analysis***

1% agarose was prepared by mixing 0.8g agarose with 80ml 1×TAE buffer and heating them in the microwave oven to fully dissolve the agarose. Then 8ul Solargel Red (Solarbio) was added in the solution. When the gel is cooled to about 50℃, evenly pour it into the plastic tank until completely cooled. Put the plastic tank into the electrophoresis tank, and make sure it was completely soaked in TAE buffer. Mix DNA samples with sample buffer and added 20ul DNA sample to each sample hole. Connect the positive and negative electrophoresis tank, set the voltage at 100V for 30 min and started electrophoresis. The DNA bands were imaged under UV imaging system. Full uncut original gels were shown in Additional file 9.
