# Supplementary material for: circNFIB1 inhibits lymphangiogenesis and lymphatic metastasis via the miR-486-5p/PIK3R1/VEGF-C axis in pancreatic cancer
Source: Mol Cancer. 2020 May 4;19:82. doi: 10.1186/s12943-020-01205-6 (PMC7197141; doi:10.1186/s12943-020-01205-6)
Supplement: Supplementary file 4 — Additional file 4 Figure S1. The identification of the downstream targets of miR-486-5p. [file 12943_2020_1205_MOESM4_ESM.doc]

**
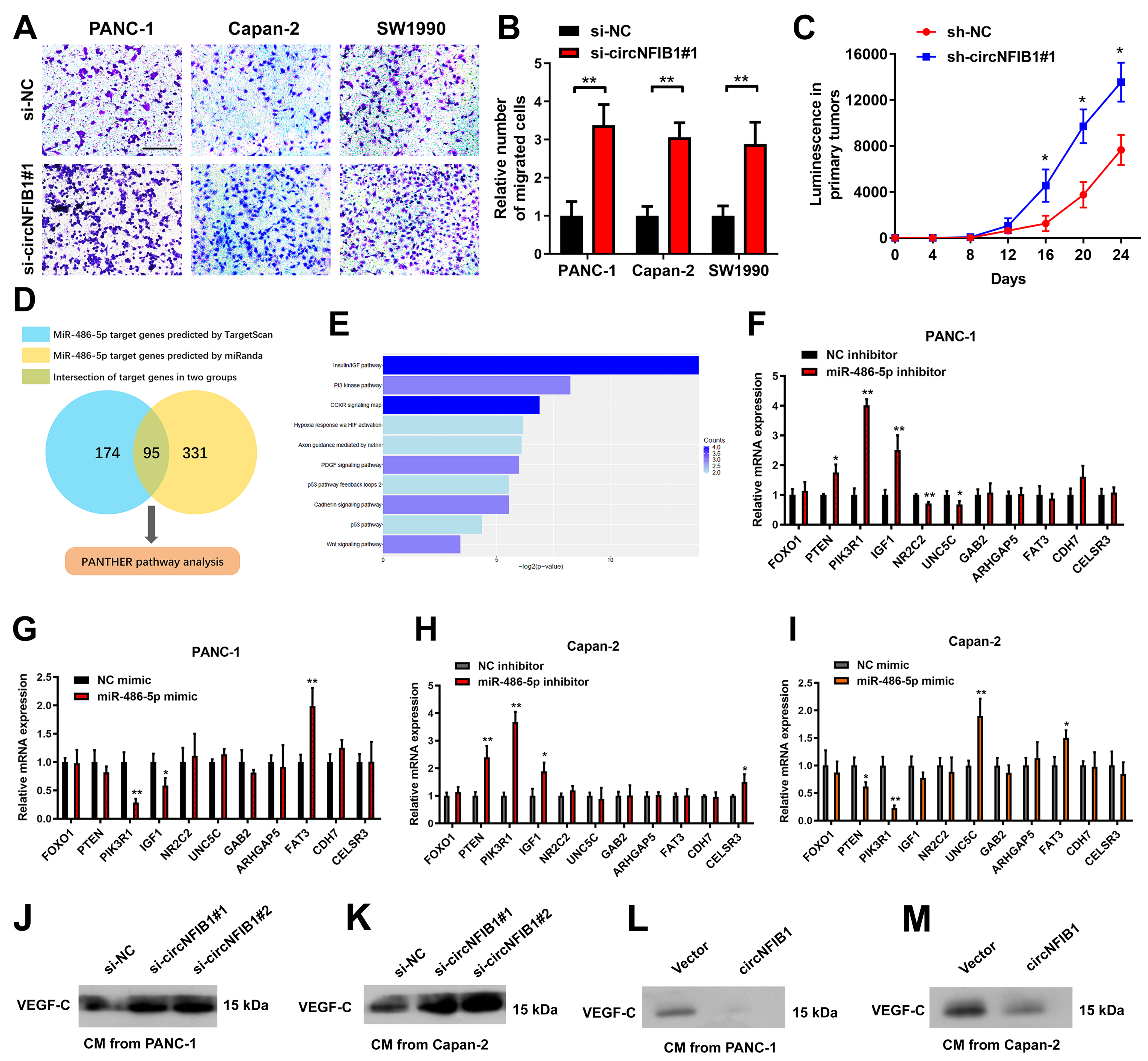
Figure S1 The identification of the downstream targets of miR-486-5p. a-b** Representative images (a) and histogram analysis (b) of Transwell assay after silencing circNFIB1 in PANC-1, Capan-2 and SW1990 cells. Scale bar: 100 μm. **c** The curve for the luminescence signal of the primary tumors was shown (n = 12 per group). **d** Schematic illustration for the prediction of miR-486-5p target genes. **e** The top 10 enriched pathways for predicted target genes of miR-486-5p were analyzed by PANTHER database. **f-i** qRT-PCR analysis of the indicated genes expression in miR-486-5p-silencing (f and h) or miR-486-5p-overexpressing (g and i) PDAC. **j-m** Western blot analysis of VEGF-C expression in culture media from PDAC after silencing (j and k) or overexpressing (l and m) circNFIB1. Significance level was assessed using two-tailed Student *t*-tests and ANOVA followed by Dunnett's tests for multiple comparison. Figures with error bars showed the standard deviations of three independent experiments. **p* < 0.05 and ***p* < 0.01.
