# Supplementary material for: circNFIB1 inhibits lymphangiogenesis and lymphatic metastasis via the miR-486-5p/PIK3R1/VEGF-C axis in pancreatic cancer
Source: Mol Cancer. 2020 May 4;19:82. doi: 10.1186/s12943-020-01205-6 (PMC7197141; doi:10.1186/s12943-020-01205-6)
Supplement: Supplementary file 5 — Additional file 5 Table S3. Univariate and multivariate analyses of OS for circNFIB1 expression in PDAC patients. [file 12943_2020_1205_MOESM5_ESM.doc]

**Table S3. Univariate and multivariate analyses of Overall Survival (OS) for circNFIB1 expression in PDAC patients (*n* = 160).**

| **Variables** | **Univariate analysis** | | | **Multivariate analysis** | | |
| --- | --- | --- | --- | --- | --- | --- |
|  | **HR** | **95%CI** | ***p*-value^A^** | **HR** | **95%CI** | ***p*-value^A^** |
| Gender (Male vs. Female) | 0.908 | 0.629-1.311 | 0.607 |  |  |  |
| Age (＞60 vs. ≤60) | 1.315 | 0.899-1.923 | 0.158 |  |  |  |
| Differentiation (poor and moderate vs. well) | 1.140 | 0.705-1.846 | 0.593 |  |  |  |
| T stage (T3-4 vs. T1-2) | 1.048 | 0.729-1.505 | 0.801 |  |  |  |
| Lymphatic metastasis (positive vs. negative) | 2.041 | 1.391-2.996 | **0.000^**^** | 1.506 | 0.878-2.581 | 0.137 |
| TNM stage (Stage III and Stage II vs. Stage I) | 1.800 | 1.121-2.889 | **0.015^*^** | 1.036 | 0.559-1.923 | 0.910 |
| circNFIB1 expression (High vs. Low) | 0.468 | 0.324-0.676 | **0.000^**^** | 0.593 | 0.382-0.920 | **0.020^*^** |

Abbreviations: HR = hazard ratio; 95%CI =95% confidence interval; T stage =tumor stage; TNM stage = tumor node metastasis stage. ^a^ Cox regression analysis, ^*^ *p* <0.05, ^**^ *p* <0.01.
