# Supplementary material for: circNFIB1 inhibits lymphangiogenesis and lymphatic metastasis via the miR-486-5p/PIK3R1/VEGF-C axis in pancreatic cancer
Source: Mol Cancer. 2020 May 4;19:82. doi: 10.1186/s12943-020-01205-6 (PMC7197141; doi:10.1186/s12943-020-01205-6)
Supplement: Supplementary file 6 — Additional file 6 Table S4. Univariate and multivariate analyses of DFS for circNFIB1 expression in PDAC patients. [file 12943_2020_1205_MOESM6_ESM.doc]

**Table S4. Univariate and multivariate analyses of Disease-free Survival (DFS) for circNFIB1 expression in PDAC patients (*n* = 160)**

| **Variables** | **Univariate analysis** | | | **Multivariate analysis** | | |
| --- | --- | --- | --- | --- | --- | --- |
|  | **HR** | **95%CI** | ***p*-value^A^** | **HR** | **95%CI** | ***p*-value^A^** |
| Gender (Male vs. Female) | 0.885 | 0.626-1.249 | 0.486 |  |  |  |
| Age (＞60 vs. ≤60) | 1.269 | 0.888-1.813 | 0.190 |  |  |  |
| Differentiation (poor and moderate vs. well) | 1.027 | 0.663-1.589 | 0.906 |  |  |  |
| T stage (T3-4 vs. T1-2) | 1.149 | 0.817-1.617 | 0.424 |  |  |  |
| Lymphatic metastasis (positive vs. negative) | 1.872 | 1.305-2.686 | **0.001^**^** | 1.339 | 0.811-2.210 | 0.254 |
| TNM stage (Stage III and Stage II vs. Stage I) | 1.738 | 1.115-2.709 | **0.015^*^** | 1.086 | 0.613-1.924 | 0.778 |
| circNFIB1 expression (High vs. Low) | 0.485 | 0.343-0.688 | **0.000^**^** | 0.587 | 0.386-0.895 | **0.013^*^** |

Abbreviations: HR = hazard ratio; 95%CI =95% confidence interval; T stage =tumor stage; TNM stage = tumor node metastasis stage. ^a^ Cox regression analysis, ^*^ *p* <0.05, ^**^ *p* <0.01.
