# Supplementary material for: circNFIB1 inhibits lymphangiogenesis and lymphatic metastasis via the miR-486-5p/PIK3R1/VEGF-C axis in pancreatic cancer
Source: Mol Cancer. 2020 May 4;19:82. doi: 10.1186/s12943-020-01205-6 (PMC7197141; doi:10.1186/s12943-020-01205-6)
Supplement: Supplementary file 7 — Additional file 7 Table S5. Antibodies used in the experiments. [file 12943_2020_1205_MOESM7_ESM.doc]

**Table S5. Antibodies used in the experiments.**

| **Product** | **Source** | **No. of Catalogue** |
| --- | --- | --- |
| **Primary antibody:** |  |  |
| ***Western blot:*** |  |  |
| anti-β-actin | Sigma-Aldrich | A5441 |
| anti-PIK3R1 (p85α) | Cell Signaling Technology | 13666S |
| anti-Akt | Cell Signaling Technology | 9272S |
| anti-p-Akt | Cell Signaling Technology | 13038S |
| anti-GSK3β | Cell Signaling Technology | 12456S |
| anti-p-GSK3β | Cell Signaling Technology | 14630S |
| ***IHC:*** |  |  |
| anti-LYVE-1 | Abcam | ab218535 |
| **Secondary antibody:** |  |  |
| ***Western blot:*** |  |  |
| anti-rabbit IgG-HRP | Cell Signaling Technology | 7074 |
| anti-mouse IgG-HRP | Cell Signaling Technology | 7076 |
| ***IHC:*** |  |  |
| anti-rabbit IgG-HRP | Proteintech | SA00001-15 |
| anti-mouse IgG-HRP | Proteintech | SA00001-1 |
