# Supplementary figures and images for: circNFIB1 inhibits lymphangiogenesis and lymphatic metastasis via the miR-486-5p/PIK3R1/VEGF-C axis in pancreatic cancer
Source: Mol Cancer. 2020 May 4;19:82. doi: 10.1186/s12943-020-01205-6 (PMC7197141; doi:10.1186/s12943-020-01205-6)

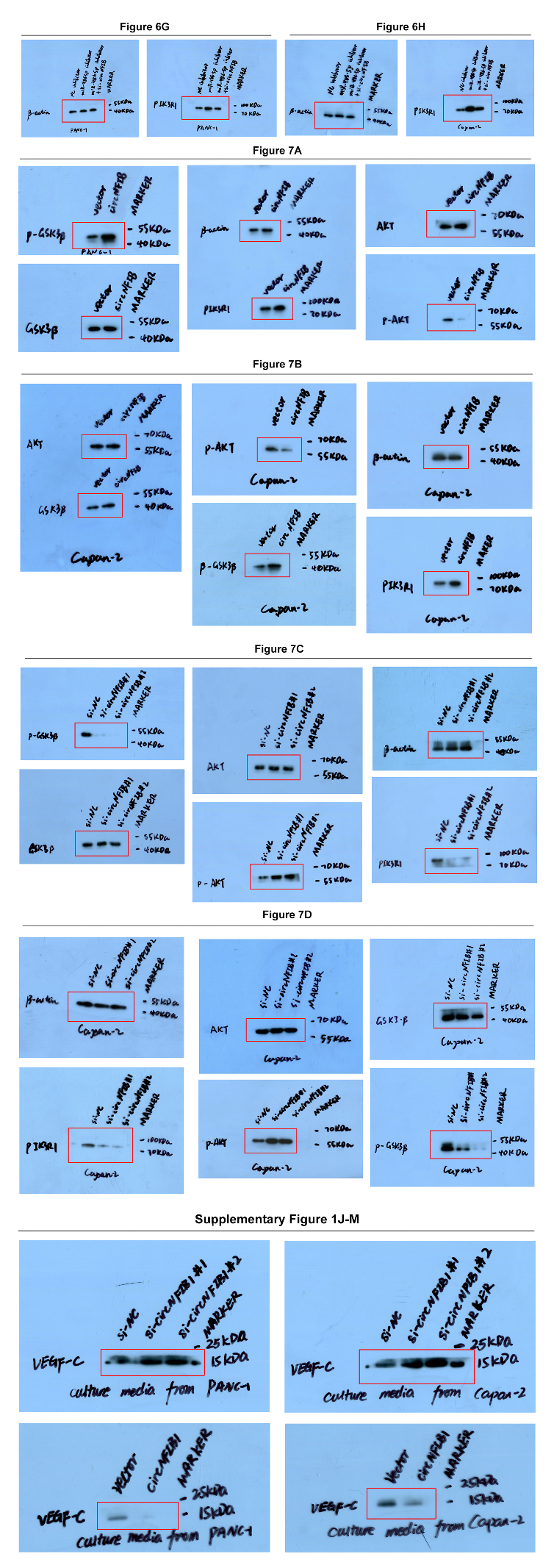


**Figure S2 Full uncut original pictures.**

Supplement: Supplementary file 8 — Additional file 8 Figure S2. Full uncut original pictures. [file 12943_2020_1205_MOESM8_ESM.docx]
