# Supplementary material for: circNFIB1 inhibits lymphangiogenesis and lymphatic metastasis via the miR-486-5p/PIK3R1/VEGF-C axis in pancreatic cancer
Source: Mol Cancer. 2020 May 4;19:82. doi: 10.1186/s12943-020-01205-6 (PMC7197141; doi:10.1186/s12943-020-01205-6)
Supplement: Supplementary file 9 — Additional file 9 Figure S3. Full uncut original gels. [file 12943_2020_1205_MOESM9_ESM.docx]

**Figure S3.** Full uncut original gels.
